# Zeeman splitting and dynamical mass generation in Dirac semimetal ZrTe_5_

**DOI:** 10.1038/ncomms12516

**Published:** 2016-08-12

**Authors:** Yanwen Liu, Xiang Yuan, Cheng Zhang, Zhao Jin, Awadhesh Narayan, Chen Luo, Zhigang Chen, Lei Yang, Jin Zou, Xing Wu, Stefano Sanvito, Zhengcai Xia, Liang Li, Zhong Wang, Faxian Xiu

**Affiliations:** 1 State Key Laboratory of Surface Physics, Fudan University, Shanghai 200433, China; 2 Department of Physics, Fudan University, Shanghai 200433, China; 3 Collaborative Innovation Center of Advanced Microstructures, Nanjing 210093, China; 4 Wuhan National High Magnetic Field Center, Huazhong University of Science and Technology, Wuhan 430074, China; 5 School of Physics, AMBER and CRANN Institute, Trinity College, Dublin 2, Ireland; 6 Department of Physics, University of Illinois at Urbana—Champaign, Urbana, Illinois 61801, USA; 7 Shanghai Key Laboratory of Multidimensional Information Processing, Department of Electrical Engineering, East China Normal University, Shanghai 200241, China; 8 Materials Engineering, The University of Queensland, Brisbane, Queensland 4072, Australia; 9 Centre for Microscopy and Microanalysis, The University of Queensland, Brisbane, Queensland 4072, Australia; 10 Institute for Advanced Study, Tsinghua University, Beijing 100084, China; 11 Collaborative Innovation Center of Quantum Matter, Beijing 100871, China

## Abstract

Dirac semimetals have attracted extensive attentions in recent years. It has been theoretically suggested that many-body interactions may drive exotic phase transitions, spontaneously generating a Dirac mass for the nominally massless Dirac electrons. So far, signature of interaction-driven transition has been lacking. In this work, we report high-magnetic-field transport measurements of the Dirac semimetal candidate ZrTe_5_. Owing to the large *g* factor in ZrTe_5_, the Zeeman splitting can be observed at magnetic field as low as 3 T. Most prominently, high pulsed magnetic field up to 60 T drives the system into the ultra-quantum limit, where we observe abrupt changes in the magnetoresistance, indicating field-induced phase transitions. This is interpreted as an interaction-induced spontaneous mass generation of the Dirac fermions, which bears resemblance to the dynamical mass generation of nucleons in high-energy physics. Our work establishes Dirac semimetals as ideal platforms for investigating emerging correlation effects in topological matters.

## Abstract (web summary)

It has been predicted that the presence of strong electronic correlations may generate new phases in materials with topologically non-trivial band structure. Here, the authors demonstrate the generation of Dirac mass in the correlated Dirac semimetal candidate ZrTe_5_ under high magnetic fields.

## Graphical abstract

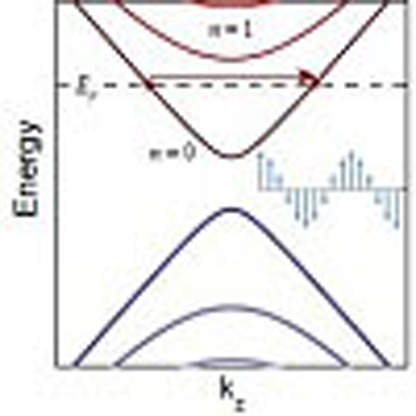

In the past few decades, transition-metal pentatelluride ZrTe_5_ has attracted considerable attentions for its mysterious temperature anomaly^1^,^2^. Charge density wave was believed to be the origin of the anomalous peak in the temperature-dependent resistance but later it was excluded by experiments^2^. At the same time, both theory^3^ and experiments^4^,^5^,^6^ demonstrated that the band structure of ZrTe_5_ is very complicated with multiple bands contributing to the electronic properties. Both single-frequency^5^,^6^ and multi-frequency^4^ Shubnikov–de Haas (SdH) oscillations were reported in ZrTe_5_, suggesting a strong dependency of the electron states on the Fermi energy, *E*_F_, in the band structure.

Recently, this material was reinvestigated as a candidate of Dirac semimetal^7^. A linear energy–momentum dispersion of the electronic structure in ZrTe_5_ was demonstrated by angle-resolved photoemission spectroscopy (ARPES)^8^ and optical spectroscopy measurements^9^,^10^. The negative magnetoresistance caused by chiral magnetic effect was also observed through magnetotransport^8^. These experimental evidences all suggest that ZrTe_5_ is a Dirac semimetal candidate, which is similar to other Dirac semimetals^11^,^12^,^13^,^14^,^15^,^16^ with extremely large magnetoresistance^17^,^18^,^19^ and the negative magnetoresistance^8^,^20^,^21^,^22^ possibly induced by the chiral anomaly. Although the single-particle physics of Dirac semimetals, including ZrTe_5_ is under intense study, the many-body correlation effects are much less investigated. A high magnetic field would significantly enhance the density of states near the Fermi level, thus effectively amplifying the correlation effects. It is therefore highly desirable to investigate the behaviour of ZrTe_5_ in the high-magnetic field regime. Possible phase transitions in high-magnetic fields have been reported in semimetallic graphite and bismuth^23^,^24^,^25^,^26^,^27^,^28^,^29^, which, however, are not ideal Dirac semimetals. For Dirac and Weyl semimetals, it has been theoretically suggested that a high-magnetic field may induce the dynamical mass generation^30^,^31^,^32^,^33^,^34^, namely, a Dirac mass is spontaneously generated by interaction effects. Depending on material details, the Dirac mass can manifest itself as charge density wave^30^,^33^,^35^, spin density wave^31^ or nematic state^34^. Although the mass generation has been observed at the surface of two-dimensional (2D) topological crystalline insulators^36^,^37^, so far there is no clear evidence of its occurrence in three-dimensional bulk Dirac materials, despite its closer resemblance to that occurring in particle physics^38^. Moreover, dynamical mass generation in three-dimensional Dirac semimetals hosts a number of unusual phenomena absent in two dimensions, for instance, it is expected that the topological dislocations associated with the dynamically generated mass may possess chiral modes^35^,^39^,^40^, holding promises to dissipationless transport inside three-dimensional bulk materials.

Here we report systematic transport measurements of single-crystal ZrTe_5_ under extremely large magnetic field. Our samples show good crystalline quality and exhibit sufficiently high electron mobility at low temperatures, enabling the observation of SdH oscillations under a relatively small magnetic field. By tilting the field direction, we mapped the morphology of the detected Fermi surface and examined the topological property of ZrTe_5_. The extracted band parameters suggest ZrTe_5_ to be a highly anisotropic material. Remarkably, with a weak magnetic field the spin degeneracy is lifted, generating a pronounced Zeeman splitting. Furthermore, by taking advantage of the tiny Fermi surface, this material can be driven into its quantum limit regime within 20 T, where all the carriers are confined in the lowest Landau level. Under the magnetic field, we observe sharp peaks in the resistivity, which are naturally explained as dynamical mass generation coming from Fermi surface nesting. The generated Dirac mass endows an energy gap to the nominally massless Dirac electrons, causing sharp increase in the resistivity. These findings not only suggest ZrTe_5_ a versatile platform for searching novel correlated states in Dirac semimetal but also show the possibility on field-controlled novel symmetry-breaking phases manifesting the Dirac mass in the study of Dirac and Weyl semimetals.

## Results

### Growth and hall-effect measurements

**Figure 1:**
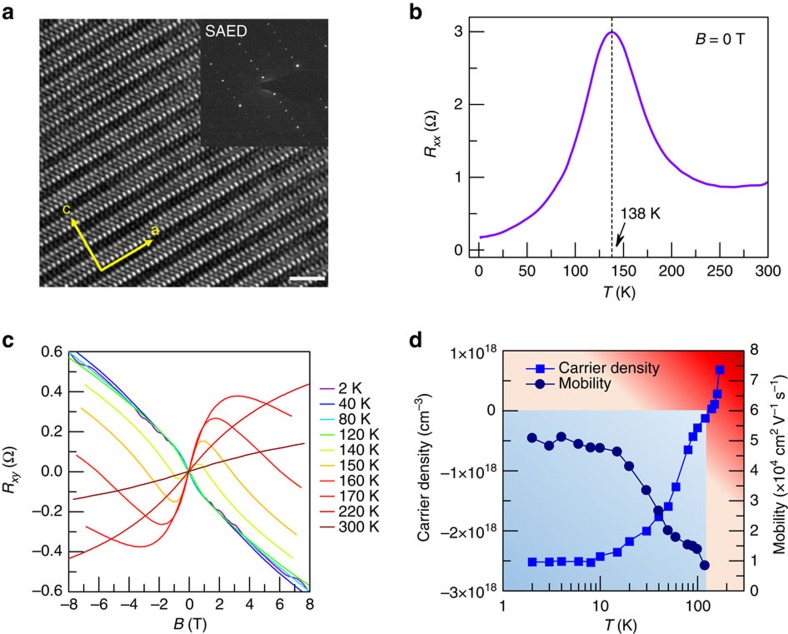
Crystal structure and Hall effect measurements of ZrTe_5_. (**a**) An HRTEM image of ZrTe_5_ with an inset selected area electron diffraction (SAED) pattern, showing the layer normal along the *b* axis. The white scale bar corresponds to 2 nm. (**b**) Temperature-dependent resistance under zero magnetic field. An anomalous resistance peak occurs at *T*∼138 K. (**c**) Temperature-dependent Hall resistance of ZrTe_5_. The nonlinear Hall slopes at both low temperature and high temperature demonstrate the multi-carrier transport in ZrTe_5_. (**d**) The temperature-dependent mobility and carrier density of the dominant carriers. A transition of electron- to hole-dominated transport is observed around the temperature of the anomalous resistance peak. The graduated background represents the amount and type of carriers, blue for holes and red for electrons.

ZrTe_5_ single crystals were grown by chemical vapour transport with iodine as reported elsewhere^41^. The bulk ZrTe_5_ has an orthorhombic layered structure with the lattice parameters of *a*=0.38 nm, *b*=1.43 nm, *c*=1.37 nm and a space group of *Cmcm* (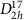)^7^. The ZrTe_5_ layers stack along the *b* axis. In the *a*–*c* plane, ZrTe_3_ chains along *a* axis are connected by Te atoms in the *c* axis direction. Figure 1a is a typical high-resolution transmission electron microscopy (HRTEM) image taken from an as-grown layered sample, from which the high crystalline quality can be demonstrated. The inset selected area electron diffraction pattern together with the HRTEM image confirms that the layer normal is along the *b* axis.

The temperature dependence of the resistance *R*_*xx*_ and Hall effect measurements provide information on the electronic states of a material in a succinct way. We first carried out regular transport measurements to extract the fundamental band parameters of as-grown ZrTe_5_ crystals. In a Hall bar sample, the current was applied along the *a* axis and the magnetic field was applied along the *b* axis (the stacking direction of the ZrTe_5_ layers). Figure 1b shows the temperature dependence of the resistance *R*_*xx*_ of ZrTe_5_ under the zero field. An anomalous peak, the unambiguous hallmark of ZrTe_5_ (ref. 4), emerges at around 138 K and is ascribed to the temperature-dependent Fermi energy shift of the electronic band structure^42^. The Hall effect measurements provide more information on the charge carriers responsible for the transport. The Hall coefficient changes sign around the anomaly temperature, implying the dominant charge carriers changing from holes to electrons (Fig. 1c). The nonlinear Hall signal suggests a multi-carrier transport at both low and high temperatures, which is also confirmed by the Kohler's plot and our first-principles electronic structure calculations (Supplementary Note 1 and Supplementary Figs 1–5). For convenience, a two-carrier transport model^43^,^44^ is adopted to estimate the carrier density and mobility. The dominant electron exhibits an ultrahigh mobility of around 50,000 cm^2^ V^−1^ s^−1^ at low temperature, which leads to strong SdH oscillations as we will discuss later. Around the temperature of the resistance anomaly, the electron carrier density has already decayed to almost one-tenth of that at low temperature, and finally holes become the majority carriers at *T*>138 K (Fig. 1d). Detailed analysis of the two-carrier transport is described in Supplementary Notes 1 and 2 and in Supplementary Figs 1–8.

### Fermi surface and quantum oscillations analysis

**Figure 2:**
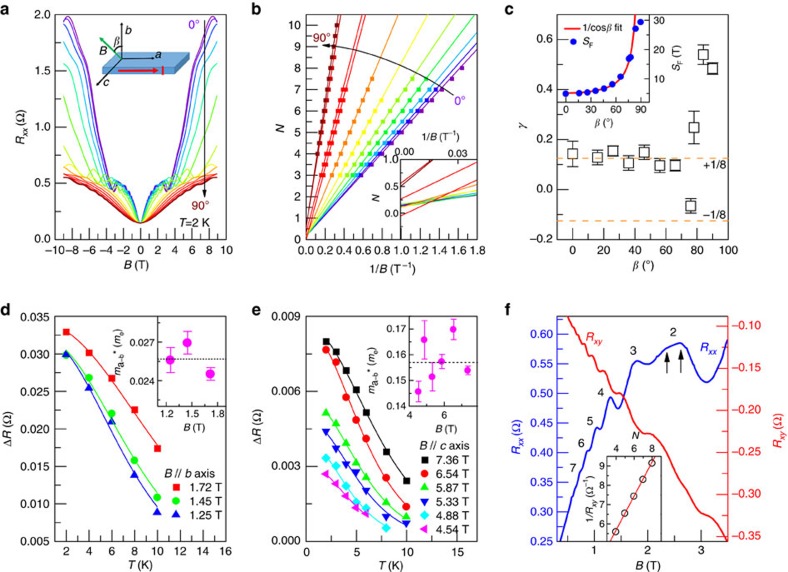
Angular MR and SdH oscillations of ZrTe_5_. (**a**) Angular MR of ZrTe_5_. The inset shows the geometry of external magnetic field. (**b**) Landau fan diagram of arbitrary angle in **a**. Inset: Zoom-in view of the intercept on *y* axis. (**c**) The angular-dependent intercept of Landau fan diagram in **b**. Inset: angular-dependent oscillation frequency. The error bars were generated from the linear fitting process in the Landau fan diagrams. (**d**,**e**) The effective mass of ZrTe_5_ when the magnetic field is applied along *b* axis and *c* axis, respectively. The error bars were generated from the fitting process. (**f**) The quantum oscillations of *R*_*xx*_ and quantized plateaus in *R*_*xy*_.

Elaborate measurements of angle-dependent magnetoresistance (MR) provide further insight into the band-topological properties of ZrTe_5_. A different external magnetic field geometry has been exploited to detect the Fermi surface at 2 K, as shown in Fig. 2. When the magnetic field *B*>0.5T is applied along the *b* axis, clear quantum oscillations can be identified, indicating a high mobility exceeding 20,000 cm^2^ V^−1^ s^−1^. The MR ratio 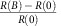 is around 10 (here *R*(*B*) is the resistance under magnetic field *B* and *R*(0) is the resistance under zero field), lower than previous results^8^ on account of different Fermi level positions. As the magnetic field is tilted away from the *b* axis, the MR damps with the law of cosines, suggesting a quasi-2D nature and a highly anisotropic Fermi surface with the cigar/ellipsoid shape. This is reasonable for a layered material^45^. A Landau fan diagram of arbitrary angle (Fig. 2b) is plotted to extract the oscillation frequency *S*_F_ and Berry's phase Φ_B_ according to the Lifshitz–Onsager quantization rule^46^: 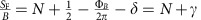, where *N* is the Landau level index, *S*_F_ is obtained from the slope of Landau fan diagram and *γ* is the intercept. For Dirac fermions, a value of |*γ*| between 0 and 1/8 implies a non-trivial *π* Berry's phase^46^, whereas a value of around 0.5 represents a trivial Berry's phase. Here the integer indices denote the Δ*R*_*xx*_ peak positions in 1/*B*, while half integer indices represent the Δ*R*_*xx*_ valley positions. To avoid the influence from the Zeeman effect, here we only consider the *N*≥3 Landau levels. With the magnetic field along the *b* axis, the Landau fan diagram yields an intercept *γ* of 0.14±0.05, exhibiting a non-trivial Berry's phase for the detected Fermi surface. At the same time, *S*_F_ shows a small value of 4.8 T, corresponding to a tiny Fermi area of 4.6 × 10^−4^ Å^−2^. The system remains in the non-trivial Berry's phase as long as 0≤*β*≤70° (Fig. 2b inset and Fig. 2c). We have also obtained the angular dependence of *S*_F_ as illustrated in the inset of Fig. 2c where a good agreement with a 1/cos*β* relationship is reached, confirming a quasi-2D Fermi surface. However, as the magnetic field is rotated towards the *c* axis (*β*>70°), the Berry's phase begins to deviate from the non-trivial and finally turns to be trivial when *B* is along the *c* axis (Fig. 2b inset, the two dark-red curves with intercept of ∼0.5). Meanwhile, the oscillation frequency *S*_F_ deviates from the cosines law and gives a value of 29.4 T along the *c* axis (Fig. 2c inset). The Berry's phase development along with the angular-dependent *S*_F_ unveils the quasi-2D Dirac nature of ZrTe_5_ and possibly a nonlinear energy dispersion along the *c* axis. This is also confirmed by the band parameters such as the effective mass and the Fermi velocity, as described below.

A meticulous analysis of the oscillation amplitude at different angles was conducted to reveal the electronic band structure of ZrTe_5_. Following the Lifshitz–Kosevich formula^46^,^47^,^48^, the oscillation component Δ*R*_*xx*_ could be described by


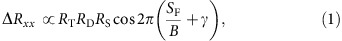


where *R*_T_, *R*_D_ and *R*_S_ are three reduction factors accounting for the phase smearing effect of temperature, scattering and spin splitting, respectively. Temperature-dependent oscillation Δ*R*_*xx*_ could be captured by the temperature smearing factor 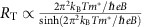, where *k*_B_ is the Boltzmann's constant, *ħ* is the reduced Plank's constant and m* is the in-plane average cyclotron effective mass. By performing the best fit of the thermal damping oscillation to the equation, the effective mass *m*_*a*–c_* (when the magnetic field is applied along the *b* axis, the Fermi surface in *a*–*c* plane is detected) is extracted to be 0.026*m*_e_, where *m*_e_ is the free electron mass (Fig. 2d). Such a small effective mass agrees well with the Dirac nature along this direction; and it is comparable to previously reported Dirac^18^,^49^,^50^ or Weyl semimetals^19^. The corresponding Fermi velocity yields a value of 5.2 × 10^5^ m s^−1^, which agrees with recent ARPES results^42^. A similar analysis gives a value of *m*_*a*–b_*=0.16*m*_e_ and *m*_*b*–c_*=0.26*m*_e_, respectively (Fig. 2e; also see Supplementary Note 3, Supplementary Figs 9–11 and Supplementary Table 1 for detailed information). Both *m*_*a*–b_* and *m*_*b*–c_* are larger than *m*_*a*–c_*; this indicates a deviation from linear dispersion of these two surfaces considering the weak interlayer coupling^7^, which is in agreement with our previous results^51^ and the reported ARPES^8^. The carrier lifetime *τ* could be obtained from the Dingle factor *R*_*D*_∼*e*^*−D*^, where 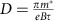. Table 1 summarizes the analysed parameters of the band structure.

Besides the obvious SdH oscillations of *R*_*xx*_, *R*_*xy*_ exhibits distinct nearly quantized plateaus, whose positions show a good alignment with the valley of *R*_*xx*_ (Fig. 2f). The value of 1/*R*_*xy*_ establishes a strict linearity of the index plot and demonstrates the excellent quantization (Fig. 2f inset), reminiscent of the bulk quantum Hall effect. A similar behaviour has been observed in several highly anisotropic layered materials, such as the heavily *n*-doped Bi_2_Se_3_ (ref. 52), *η*-Mo_4_O_11_ (ref. 53) and organic Bechgaard salt^54^,^55^,^56^. At variance with the quantum Hall effect in a 2D electron gas, the quantization of the inverse Hall resistance does not strictly correspond to the quantum conductance. In fact, because of the weak interlayer interaction^7^, bulk ZrTe_5_ behaves as a series of stacking parallel 2D electron channels with layered transport, which leads to the 2D-like magneto-transport as discussed above. The impurity or the coupling between the adjacent layers in the bulk causes the dissipation so that the *R*_*xx*_ cannot reach zero^52^. It is worth noting that the peak associated to the second Landau level in *R*_*xx*_ displays a broad feature with two small corners marked by the arrows, implying the emergence of spin splitting.

### Zeeman splitting under extremely low temperature

**Figure 3:**
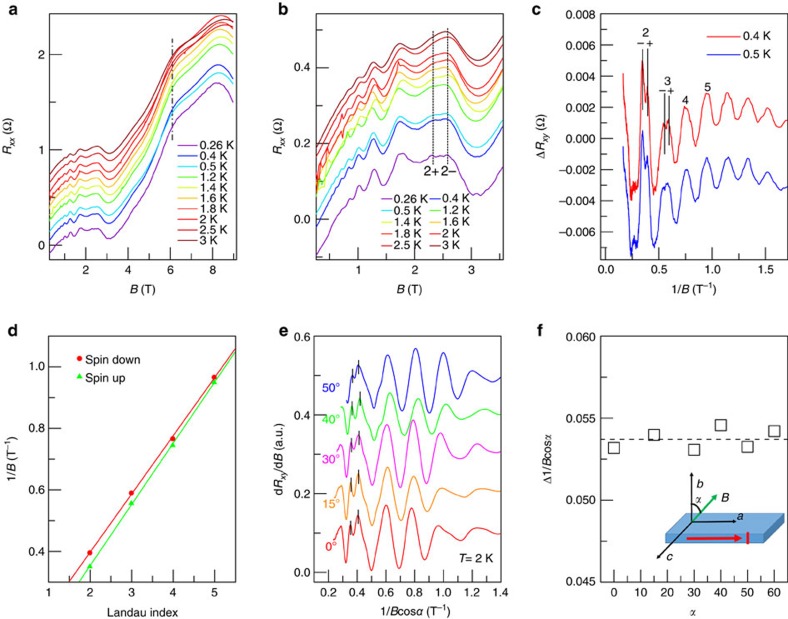
Zeeman splitting in ZrTe_5_. (**a**) MR behaviour of ZrTe_5_ at the temperature range of 0.26–3 K. (**b**) Temperature-dependent MR of ZrTe_5_. Two dashed lines are a guide to the eyes, which indicate the Zeeman splitting of the second Landau level. (**c**) The oscillation component in *R*_*xy*_ at 0.4 and 0.5 K. Sizable Zeeman splitting can be distinguished from the second and third Landau levels. (**d**) Landau fan diagram for both spin-up and spin-down electrons. (**e**) Angular dependence of the first-order differential *R*_*xy*_ versus 1/*B*cos*α*. (**f**) The spacing of Zeeman splitting in the second Landau level at different field angles. The inset shows the geometry of external magnetic field.

It is quite remarkable that the spin degeneracy can be removed by such a weak magnetic field. To investigate the spin splitting, it is necessary to further reduce the system temperature. Figure 3a shows the MR behaviour of ZrTe_5_ at 260 mK. A peak deriving from the first Landau level can be observed at ∼6 T. The second Landau level offers a better view of the Zeeman splitting because of the relatively small MR background, as marked by the dashed lines in Fig. 3b. The *R*_*xy*_ signal provides a much clearer signal: after subtracting the MR background, it reveals strong Zeeman splitting from the oscillatory component Δ*R*_*xy*_ (Fig. 3c and Supplementary Fig. 12). At 0.4 K, the fifth Landau level begins to exhibit a doublet structure with a broad feature. Under higher magnetic fields, the separation of the doublet structure increases, in particular, the second Landau level completely evolves into two peaks, indicating the complete lifting of spin degeneracy due to the Zeeman effect. To analyse the Zeeman effect occurred at such low temperature conveniently, we rearranged the spin phase factor 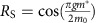
in equation (1) by the product-to-sum formula^47^ (detailed mathematical process is available in Supplementary Note 4). As a result, the oscillation component Δ*R*_*xx*_ is equivalent to the superposition of the oscillations from the spin-up and spin-down Fermi surface


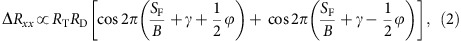


where 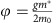 is the phase difference between the oscillations of spin-up and spin-down electrons. With this method we can estimate the *g* factor by Landau index plot for both spin ladders (Fig. 3d). This leads to the *g* factor of 21.3, in good agreement with the optical results^9^. Given such a large *g* factor, it is understandable that the Zeeman splitting could be easily observed in a relatively weak magnetic field. We have further carried out the theoretical Landau level calculations, which provides a clear insight into the Zeeman splitting as elaborated in Supplementary Note 5. In short, when the magnetic field is along the *b* axis (*z* direction), the Landau level energy eigenvalues for *n*≠0 are 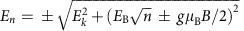, where *μ*_B_ is the Bohr magneton, and in this case *E*_*k*_=*ħv*_*z*_*k*_*z*_, 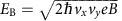 is the Landau level energy of the band bottom of the *n*=1 Landau level. Here the Landau levels are split by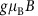, resulting in the observed Zeeman splitting.

The angular-dependence of the Zeeman splitting can provide valuable information to probe the underlying splitting mechanism. Figure 3e shows the first-order differential *R*_*xy*_ as a function of 1/*B*cos*α*. Pronounced quantum oscillations with Zeeman splitting can be unambiguously distinguished and they align well with the scale of 1/*B*cos*α*, further verifying the quasi-2D Fermi surface as mentioned before. It is noticeable that the spacing of the Zeeman splitting changes with the field angle. Generally, Zeeman splitting effect is believed to scale with the total external magnetic field so that the spacing of the splitting Landau level would not change with angle. However, in the case of ZrTe_5_, the spacing of the Zeeman splitting, normalized by *B*cos*α*, is consistent with the quasi-2D nature (Fig. 3f). The angular dependent Zeeman splitting can be attributed to the orbital contribution caused by strong spin-orbit coupling in ZrTe_5_ (ref. 7). Regarding the effect of the exchange interaction induced by an external field, we may decompose the splitting into an orbital-dependent and an orbital-independent part, where the former one depends on the shape of the band structure that leads to the angle-dependent splitting, and the latter one mainly comes from the Zeeman term that hardly contributes to any angular dependent splitting^11^. Owing to the highly anisotropic Fermi surface of ZrTe_5_ and the strong spin orbital coupling from the heavy Zr and Te atoms, the orbital effect in the *a*–*c* plane of ZrTe_5_ is significant, giving rise to a highly anisotropic *g* factor and an angular-dependent Zeeman splitting. Similar phenomena have also been observed in materials such as Cd_3_As_2_ (refs 49, 57) and Bi_2_Te_3_ (ref. 58).

### Magnetotransport under high magnetic fields

**Figure 4:**
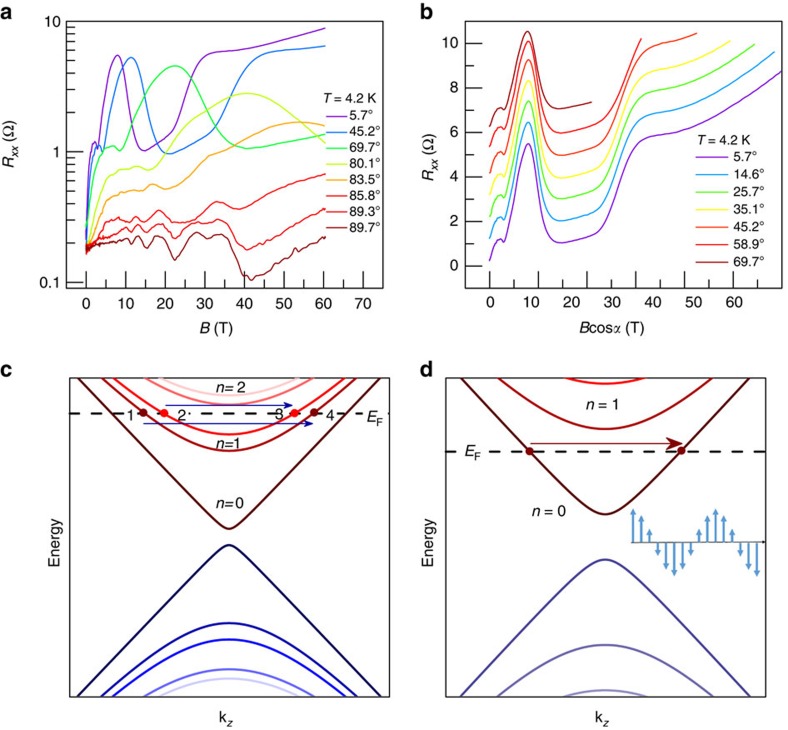
Ultra-quantum limit transport and dynamical mass generation of ZrTe_5_ at 4.2 K. (**a**) Angular-dependent MR of ZrTe_5_ at 4.2 K under high magnetic field up to 60 T. (**b**) Angular-dependent MR as a function of effective magnetic field perpendicular to *a*–*c* plane. (**c**,**d**) The Landau levels and Fermi levels for *B*≈9 and 25 T, respectively. The inset of **d** is an illustration of the spin density wave from the *n=*0 Landau level.

A high magnetic field up to 60 T was applied to drive the sample to the ultra-quantum limit to search for possible phase transitions. Figure 4a shows the angular-dependent MR of ZrTe_5_ under strong magnetic field. The measurement geometry can be found in Fig. 3f inset. Several features are immediately prominent. First, when the magnetic field is along the *a* axis, Zeeman splitting is observed, which is consistent with the theoretically solved Landau levels (Supplementary Note 5). The *g* factor along the *a* axis extracted by formula (2) is 3.19, which agrees well with the anisotropy of *g* factor discussed above (Supplementary Fig. 13). When the magnetic field is along the *c* axis, spin-splitting is hardly observed, again consistent with the theoretical expectations and the recent magneto-spectroscopy results^9^ (Supplementary Note 6 and Supplementary Fig. 14). Here the Landau level energy eigenvalues become 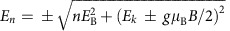, here *E*_*k*_=*ħv*_*y*_*k*_*y*_. The effect of the magnetic field is the horizontal shifting of the degenerate Landau levels by ±*gμ*_B_*B*/*ħv*_*y*_ in the *k*_*y*_ vector direction (the field direction), instead of splitting the heights of the band bottom like the case when the field is along the *a* or *b* axis, thus there is no Landau level splitting in the quantum oscillation. Second, even with an external magnetic field up to 60 T (in the quantum limit regime), the MR feature still follows the *B*cos*α* fitting, confirming once again the quasi-2D nature of ZrTe_5_ (Fig. 4b). Finally, and most importantly, a huge resistance peak emerges at around 8 T, followed by a flat valley between 12 and 22 T, then the resistance increases and forms a shoulder-like peak at ∼30 T. It should be emphasized that the amplitude of the resistance at 8 T is much larger than the amplitude of SdH oscillations, so that the signal of the first Landau level has been submerged into the anomalous peak. Only a few materials show analogous field-induced electronic instabilities, such as bismuth^23^,^24^,^26^, graphite^25^,^29^,^59^, and more recently the Weyl semimetal TaAs (ref. 60). As we have remarked, the SdH peak due to the *n*=1 Landau level merges into the anomalously large peak around *B*=8 T, which suggests that the *n*=2 Landau levels are empty and the electrons in the *n*=1 Landau level are responsible for the anomalous peak. The location of Fermi level is schematically shown in Fig. 4c. The peak can be naturally explained by the picture of dynamical mass generation (accompanied by a density wave formation, with the wave vector being the nesting vector) in the *n*=1 Landau levels, which leads to the generation of an energy gap for the electrons in these Landau levels, thus significantly enhancing the resistivity. In Fig. 4c, we illustrate one of the possibilities of nesting vectors responsible for this instability (the other possibility being two vectors connecting the Fermi momenta 1 to 3, and 2 to 4, respectively). Since the vector 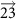 is slightly different from the vector 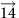 due to the Zeeman splitting, the density wave transition should also be Zeeman split, which is presumably responsible for the existence of a ‘bump' near the top of the peak in Fig. 2a.

On further increasing the magnetic field, the resistivity reaches a minimum at around *B*=14 T (Fig. 4b), which can be explained as a reentrant transition due to the crossing of the Fermi level with the band bottom of the *n*=1 Landau level. A similar phenomenon has been observed in graphite^61^. In fact, if we take a simple Bardeen–Cooper–Schrieffer model for the density wave state, we have 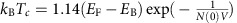, which predicts that *T*_*c*_=0 as *E*_F_ approaches *E*_B_, leading to the destruction of density waves. Here *N*(0) and *V* are the density of states at the Fermi level and the interaction parameter (of the order of several eV), respectively. As the magnetic field increases, the density wave in the first Landau level is destroyed because of such reentrant transition, consequently the resistance reduces.

Further increasing the magnetic field to *B*=25 T, the resistivity begins to increase sharply again (Fig. 4a). Since this phenomenon occurs in the ultra-quantum limit where almost all the electrons are confined in the *n*=0 Landau level, it can be explained as a dynamical mass generation in the *n*=0 Landau level. The nesting vector of this density wave transition is shown in Fig. 4d. The explicit form of the density wave is inaccessible by the transport experiments, nevertheless, we can theoretically calculate it following ref. 24. With the low-energy Hamiltonian 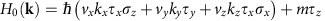, where the Pauli matrices *τ*_*x*,*y*,*z*_ refers to certain orbital degrees of freedom and *σ*_*x*,*y*,*z*_ represents the electron spin, and *m* is a mass parameter that is almost zero. The *x*, *y*, *z* axes correspond to *a*, *c*, *b* axes of the crystal, respectively. Then we have the Landau level wave functions for the zeroth Landau level:


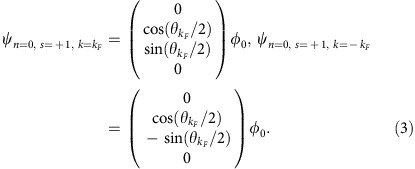


Here *θ*_*k*_ satisfies tan*θ*_*k*_=*E*_*k*_/*E*_*k*_, and *E*_*z*_=*μ*_B_*gB*/2. Inserting the wave function (3) into the *z*-component of the spin density 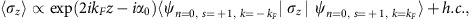 we have (Supplementary Note 7 and Supplementary Fig. 15)


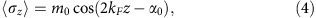


where *α*_0_ is a constant phase angle and the constant *m*_0_ can be taken as the order parameter of the density wave. For the first Landau level, the density wave also has the similar form of 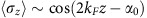. It would be interesting to demonstrate the form of density wave state directly in the future experiments such as spin-resolved scanning tunnelling microscopy. And such an exotic field-induced density wave is also confirmed in HfTe_5_, which has a similar crystal structure and physical properties as ZrTe_5_ (More details are available in Supplementary Note 8 and Supplementary Figs 16–18).

## Discussion

Finally, let us briefly compare ZrTe_5_ with the previously widely studied Dirac semimetals such as Cd_3_As_2_. In Cd_3_As_2_, it is difficult to observe such field-induced density wave transitions with ultra-high magnetic field^49^,^62^ because of their high Fermi velocity along any directions, leading to lower density of states insufficient to achieve pronounced density wave transitions or dynamical mass generation. In contrast, the strong anisotropy of ZrTe_5_ leads to an exotic layered transport and a quasi-2D Fermi surface. The consequent Fermi velocity along the *b* axis is very small, which strongly enhances the density of states when the magnetic field is along the *b* axis. Another advantage of ZrTe_5_ is the relatively tiny Fermi surface in *a*–*c* plane, making it accessible to reach the low Landau level with relatively weak magnetic field. With the strong magnetic field, the electron–electron interaction can be efficiently enhanced, amplifying the instability towards dynamical mass generation. The unambiguous Dirac feature in the *a*–*c* plane together with the highly anisotropic Fermi velocity makes ZrTe_5_ an outstanding platform to study the field-induced instabilities of Dirac fermions.

In summary, we have studied the transport properties of the newly discovered Dirac semimetal ZrTe_5_, and found signatures of the field-induced dynamical mass generation. As a quasi-2D Dirac material with small Fermi velocity along the layered direction, ZrTe_5_ is an ideal material to explore field-induced many-body effects. This study may open up a research avenue in the subject of Dirac and Weyl semimetals, namely, field-controlled symmetry-breaking phases manifesting the Dirac mass. In the future, it will also be highly interesting to search for the topological dislocations^35^,^39^,^40^ of the dynamically generated mass, which may host the dissipationless chiral modes. In a wider perspective, this study shows the possibility of investigating and engineering interaction effects in topological materials, topological semimetals in particular, by applying external fields.

## Methods

### Sample synthesis and characterizations

High-quality single crystals of ZrTe_5_ were grown via chemical vapour transport with iodine. Stoichiometric Zirconium flake (99.98%, Alfa Aesar) and Tellurium powder (99.999%, Alfa Aesar) were ground together and sealed in an evacuated quartz tube with iodine flake (99.995%, Alfa Aesar). A temperature gradient of 150 °C between 580 and 430 °C in a two-zone furnace was used for crystal growth. Typical as-grown sample has a long ribbon-like shape. HRTEM was carried out on JEM-2100F. An acceleration voltage of 200 kV was chosen to achieve enough resolution while maintaining the structure of ZrTe_5_.

### Transport measurements

The low-field magneto-transport measurements were performed in a Physical Property Measurement System by Quantum Design with a magnetic up to 9 T. The 60 T pulsed magnetic field measurements were performed at Wuhan National High Magnetic Field Center.

### Data availability

The data that support the findings of this study are available from the corresponding author on request.

## Additional information

**How to cite this article:** Liu, Y. *et al.* Zeeman splitting and dynamical mass generation in Dirac semimetal ZrTe_5_. *Nat. Commun.* 7:12516 doi: 10.1038/ncomms12516 (2016).

## Supplementary Material

Supplementary Information Supplementary Figures 1-18, Supplementary Table 1, Supplementary Notes 1-8 and Supplementary References.

## Figures and Tables

**Table 1:** Band parameters of ZrTe_5_.

Geometry	Effective mass	Frequency	Fermi area	Fermi velocity	Lifetime
m*/m_e_	*S*_F_ (T)	*A*_F_(Å^−2^)	*v*_F_ (10^5^ ms^−1^)	*τ* (ps)	
*b*–*c* plane	0.26	46.6	4.4 × 10^−3^	1.7	0.16
*a*–*c* plane	0.026	4.8	4.6 × 10^−4^	5.2	0.13
*a*–*b* plane	0.16	29.4	2.8 × 10^−3^	2.2	0.21

The band parameters, including the effective mass m*, Fermi surface *S*_F_, Fermi area *A*_F_, Fermi velocity *v*_F_ and lifetime ***τ*** can be extracted from the SdH oscillations.
