# Supplementary material for: Zeeman splitting and dynamical mass generation in Dirac semimetal ZrTe5
Source: Nat Commun. 2016 Aug 12;7:12516. doi: 10.1038/ncomms12516 (PMC4990656; doi:10.1038/ncomms12516)
Supplement: Supplementary Information — Supplementary Figures 1-18, Supplementary Table 1, Supplementary Notes 1-8 and Supplementary References. [file ncomms12516-s1.pdf]

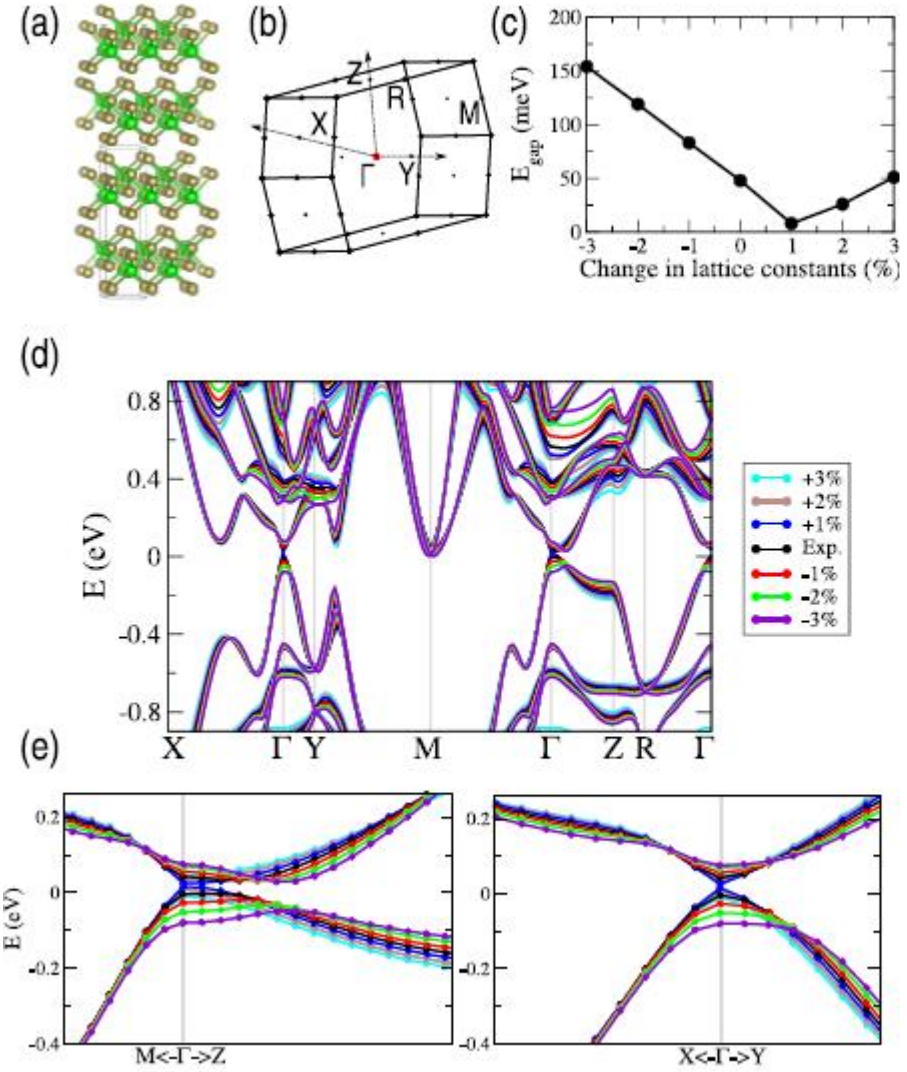

Supplementary Figure 1. $ZrTe_5$ unit cell, Brillouin zone and band structure. a, Unit cell for $ZrTe_5$, where the green spheres indicate Zr atoms, while brown spheres depict Te atoms. **b,** Brillouin zone of $ZrTe_5$, with the high-symmetry points marked. **c,** Variation of the band gap at the Brillouin zone centre with changing lattice constants. Reference experimental lattice constants are obtained from Ref. 1. **d,** Relativistic band structure of bulk $ZrTe_5$ at different values of lattice constants. Left and right panels in **e** show a zoom-in-view of the band structure along M-Γ-Z and X-Γ-Y directions, respectively.

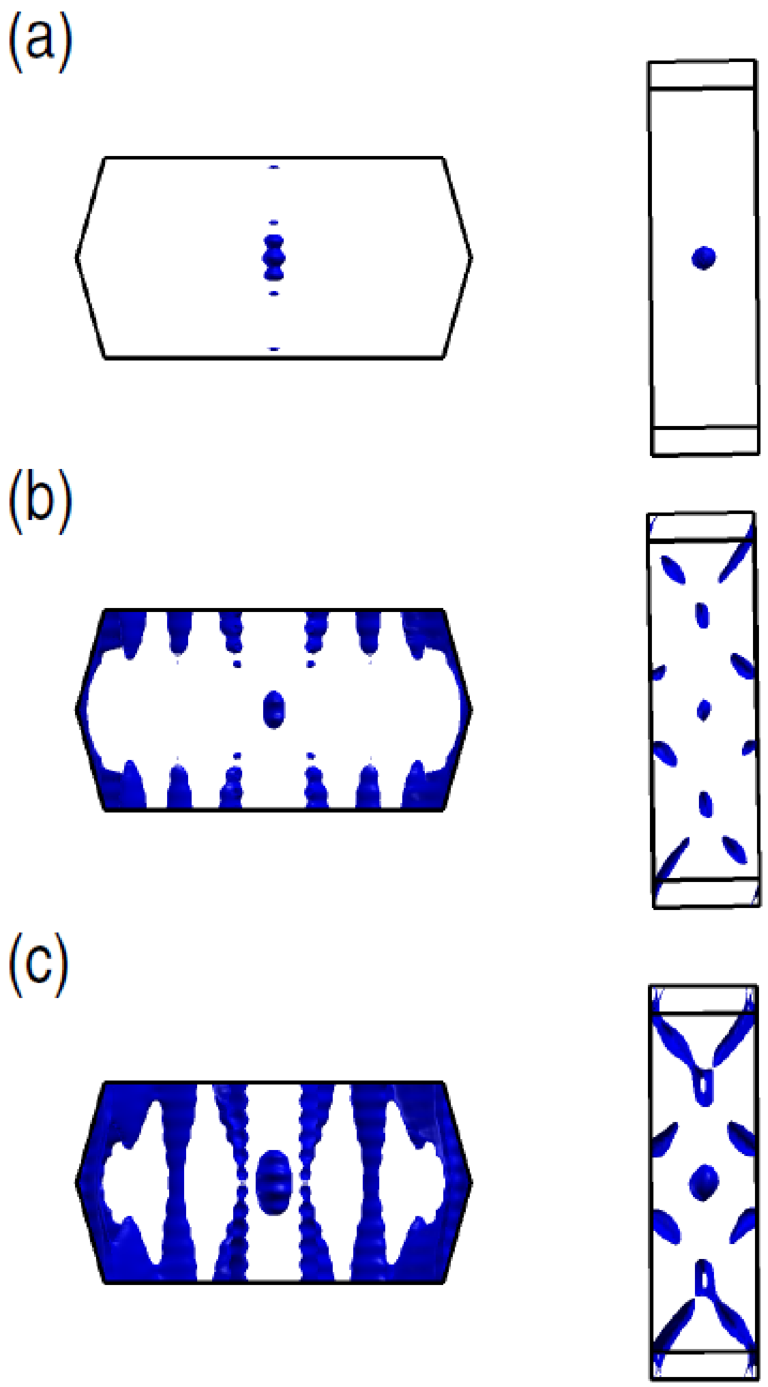

Supplementary Figure 2. Fermi surface of $ZrTe_5$. Side view (left) and top view (right) of Fermi surfaces at (a) 100 meV below the Fermi level, (b) 50 meV above the Fermi level and (c) 100 meV above the Fermi level, for $ZrTe_5$ with lattice constants chosen to be one percent greater than the reported value from Ref. 1.

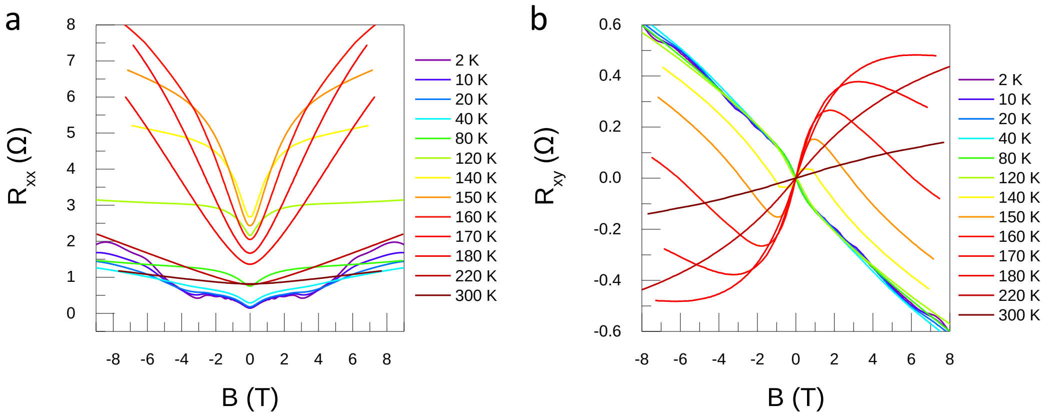

Supplementary Figure 3. The temperature-dependent magneto-transport of $ZrTe_5$. a, Temperature-dependent MR of $ZrTe_5$. The MR exhibits SdH oscillations at 2~20 K. As the temperature increases (40~150 K), it shows a saturation under high magnetic field. When the temperature reaches 160~300 K, the MR gives a parabolic behavior. **b,** Temperature-dependent Hall resistance of $ZrTe_5$. At low temperatures, it generates negative slope showing the electron-dominated transport. Around the transition temperature, the Hall slope changes sign with both electrons and holes contributing to the transport (two-carrier transport). At high temperatures, the positive Hall slope reveals a hole-dominated transport.

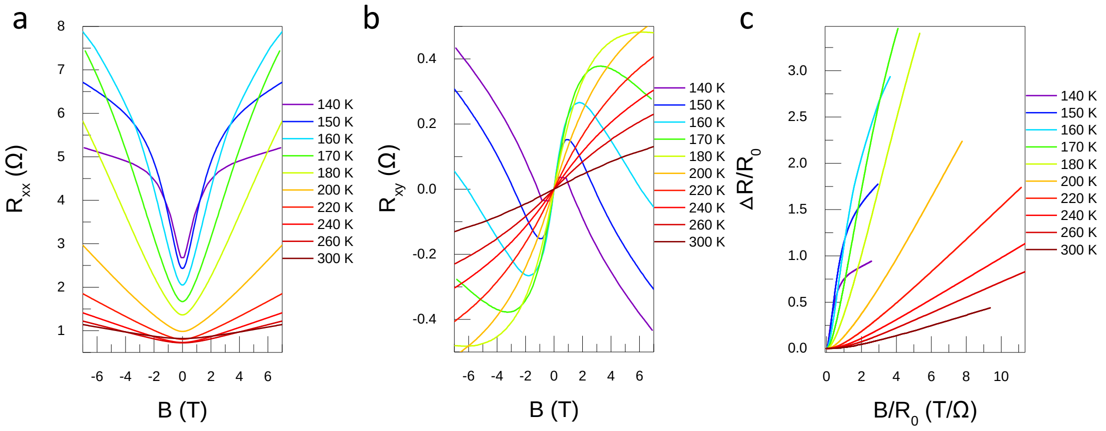

Supplementary Figure 4. Temperature-dependence of multi-carrier transport of $ZrTe_5$ around the anomalous resistivity peak. a-b, Temperature dependence of MR and Hall resistance at high temperatures, respectively. The nonlinear Hall signal reveals the multi-carrier transport. **c,** Temperature dependence of the Kohler's plot.

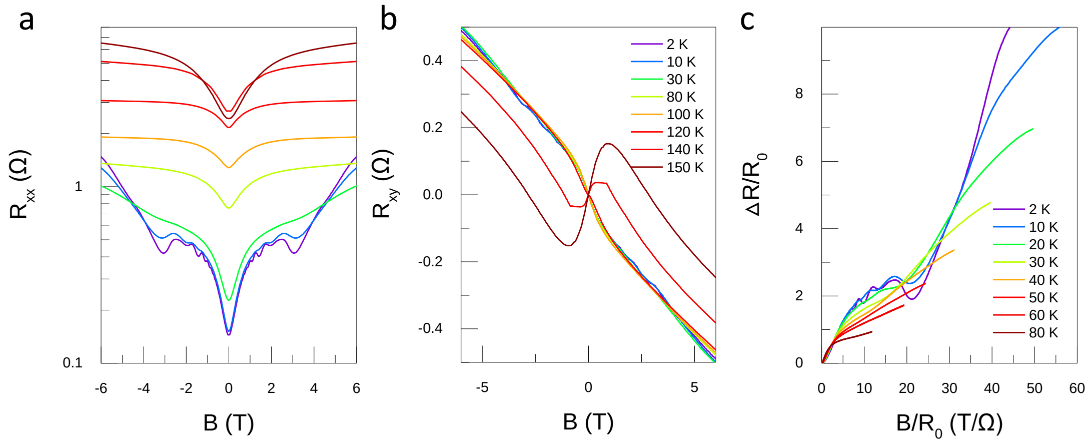

Supplementary Figure 5. Multi-carrier transport of $ZrTe_5$ at low temperatures. a-b, Temperature dependence of MR and Hall resistance at low temperatures, respectively. Electrons dominate the transport with negative Hall slope. **c,** Temperature dependence of the Kohler's plot.

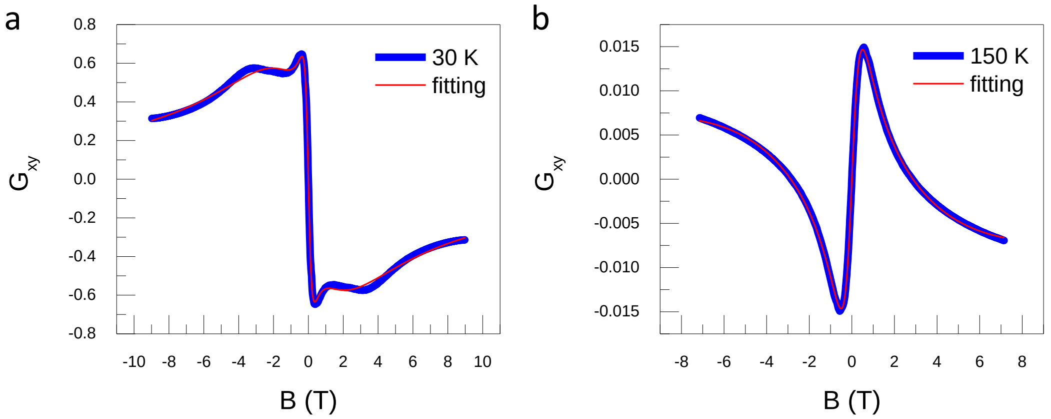

Supplementary Figure 6. Two-carrier transport fitting of the G_{xy} at 30 K and 150 K. The blue curves are the experimental results while the red curves are the fitting curves.

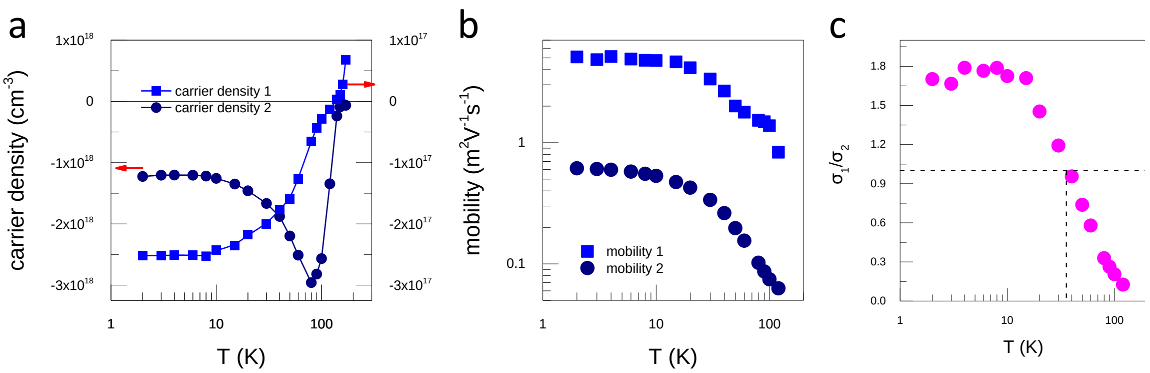

Supplementary Figure 7. Temperature-dependence of carrier density and mobility. **a,** Temperature-dependence of carrier density for two types of electrons. **b,** Temperature-dependent mobility. **c,** Temperature-dependent conductance ratio between two types of electrons.

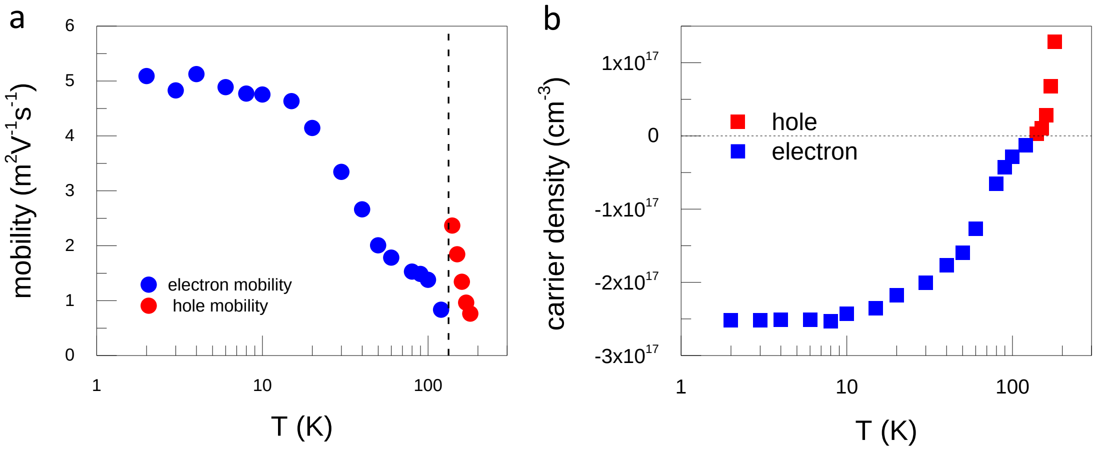

Supplementary Figure 8. Temperature dependence of carrier density (a) and mobility (b) of the high mobility carrier. The *p-n* transition occurs at 138 K, accompanied by an anomalous resistance peak.

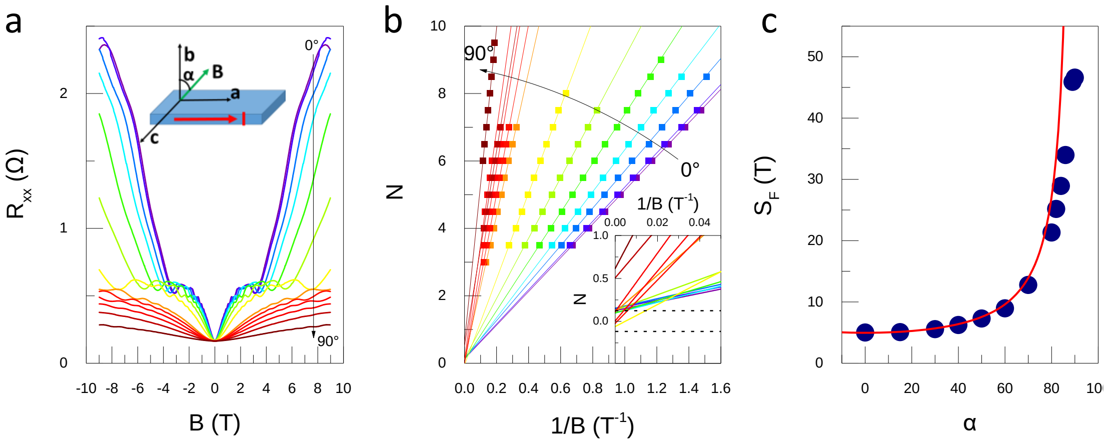

Supplementary Figure 9. Magnetoresistance of $ZrTe_5$ with the rotated magnetic field in *a-b* plane. a, Angular-dependent magnetoresistance of $ZrTe_5$ at 2 K. **b,** Landau fan diagram with different magnetic field orientation. Inset: the intercept of Landau fan diagram. **c,** Angular-dependent frequency of quantum oscillations.

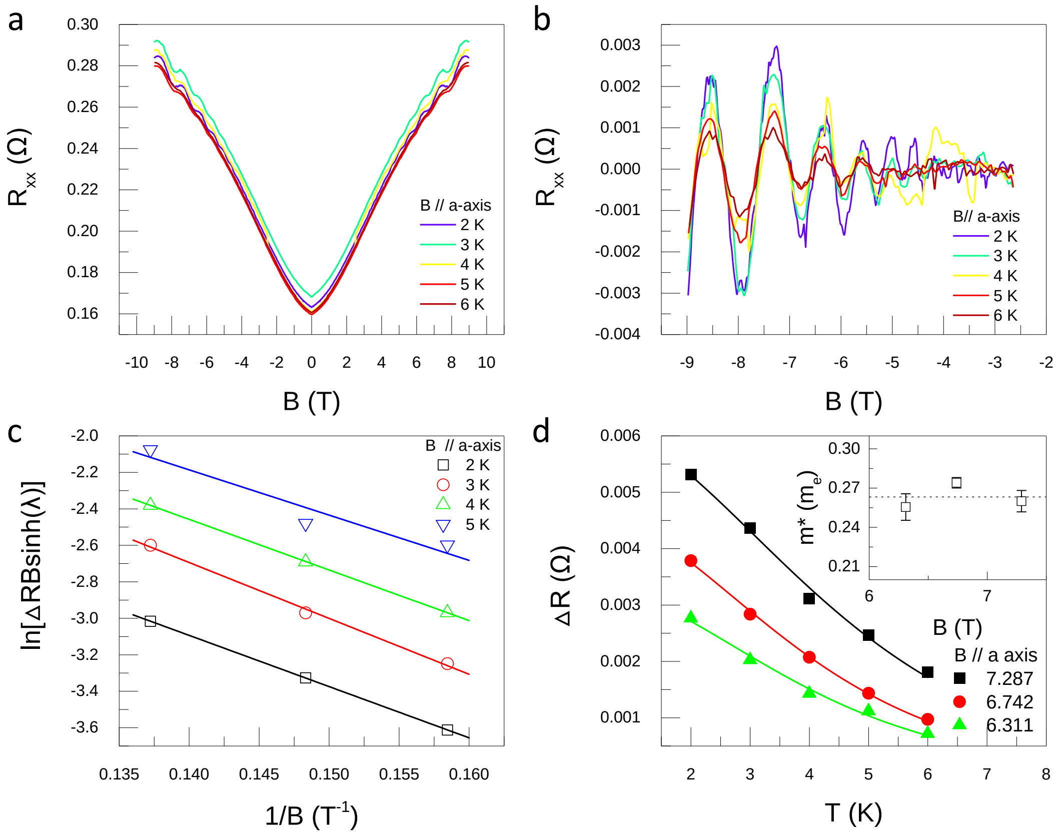

Supplementary Figure 10. Quantum oscillations of $ZrTe_5$ with magnetic field applied along *a*-axis. a, Temperature-dependent MR of $ZrTe_5$ with magnetic field

parallel to *a*-axis. **b,** The extracted quantum oscillations of $ZrTe_5$ based on **a**. The analysis of quantum oscillations - Dingle plot - gives the quantum lifetime and effective mass **(c)** and temperature factor fitting **(d)**.

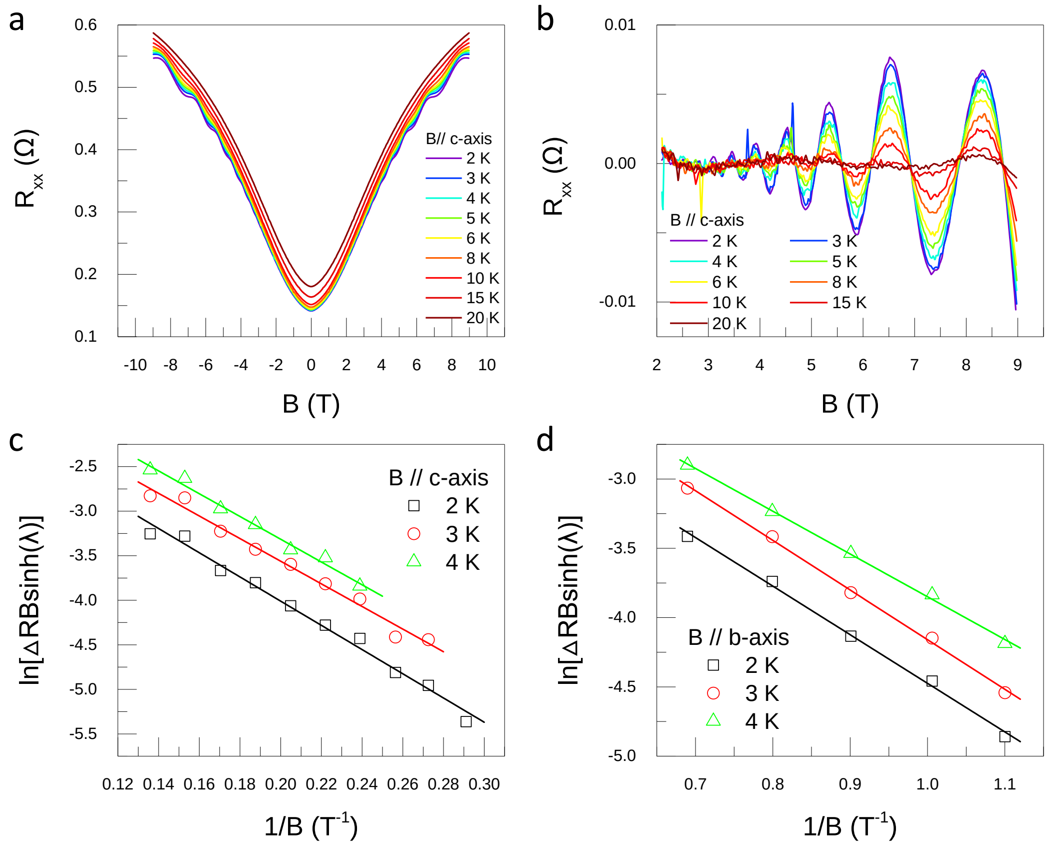

Supplementary Figure 11. Quantum oscillations of $ZrTe_5$ with magnetic field along the *c*-axis. a, Temperature-dependent MR of $ZrTe_5$ with magnetic field along the *c*-axis. **b,** The extracted SdH oscillations. **c-d,** Dingle plots of *B*//*c*-axis and b-axis, respectively.

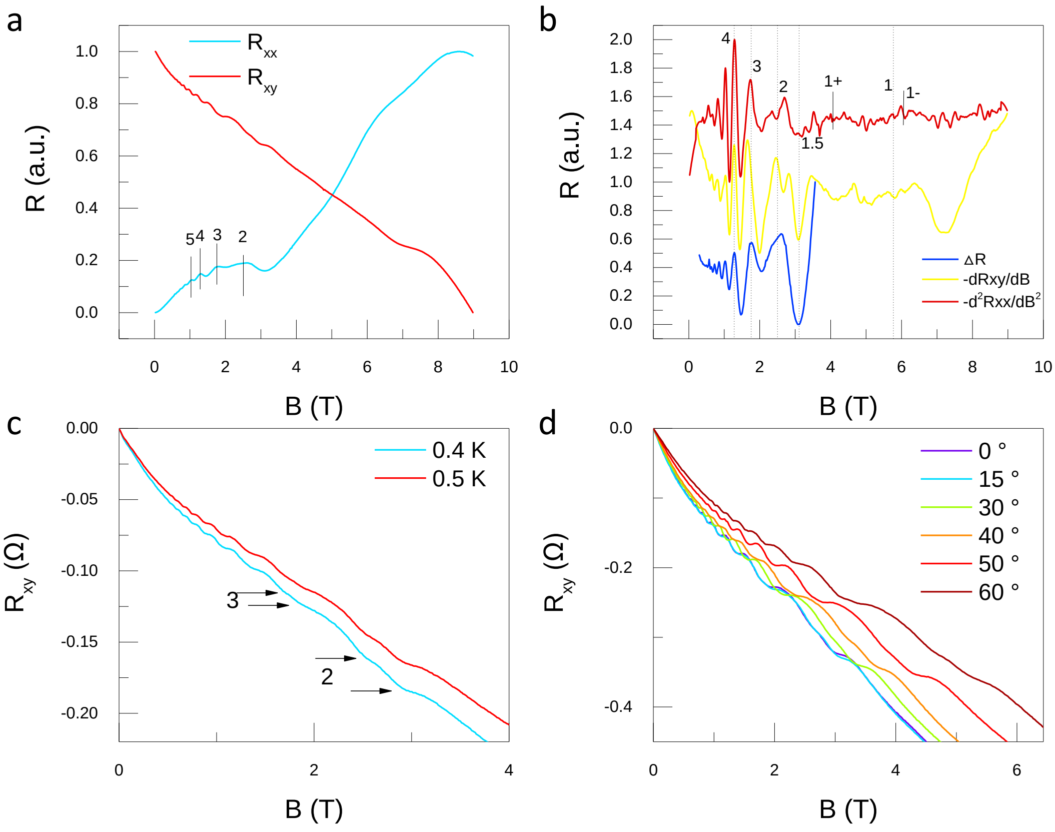

Supplementary Figure 12. Quantum oscillations and Zeeman splitting from R_{xx} and R_{xy} of $ZrTe_5$. a, Normalized MR and Hall resistance at 2 K. The solid lines mark the oscillations observed from R_{xx}. **b,** The oscillation components extracted via different approaches. **c,** The Hall resistance under 0.4 K and 0.5 K, respectively. The arrows here mark the splitting signal of 3rd and 2nd Landau levels, respectively. **d,** Angular dependent Hall resistance at 2 K. Figure 3e in the main text is extracted from this figure.

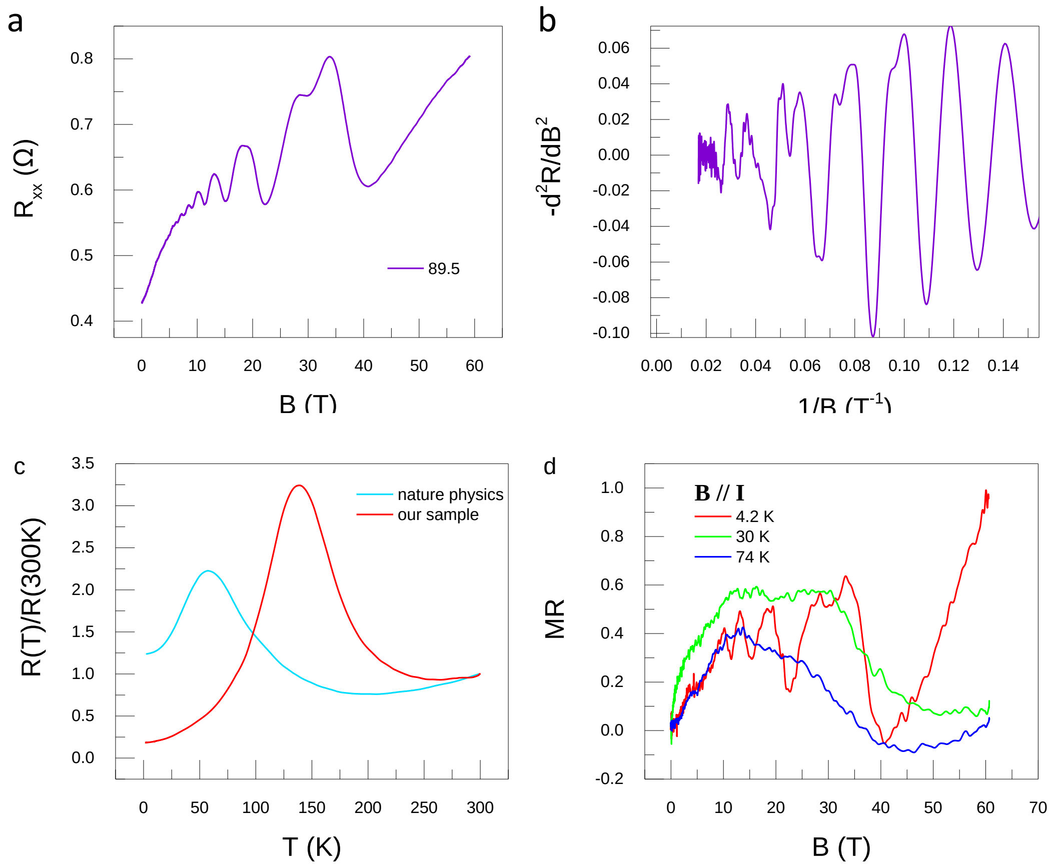

Supplementary Figure 13. MR (a) and quantum oscillations (b) of $ZrTe_5$ when magnetic field is along *a*-axis. c, Temperature-dependent residual resistance ratio of $ZrTe_5$ from nphys3648 and our manuscript. **d,** Longitudinal MR of $ZrTe_5$ at different temperatures. It shows positive MR at low temperatures with quantum oscillations. The negative MR develops as the temperature increases.

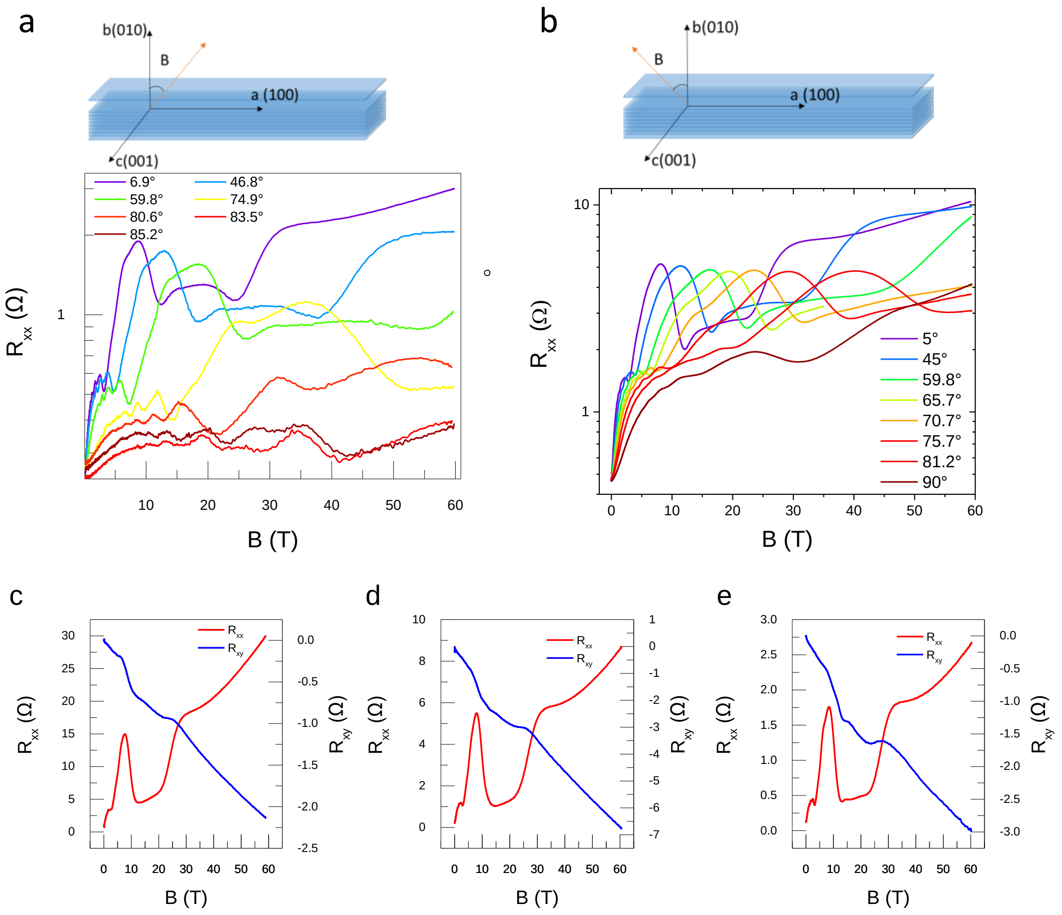

Supplementary Figure 14. Angular-dependent MR of $ZrTe_5$ and Hall resistance under high magnetic field at 4.2 K. a, Angular-dependent MR of another sample with magnetic field rotated in *a*-*b* plane. **b,** Angular-dependent MR with magnetic field rotated in *b*-*c* plane. **c-e,** R_{xx} and R_{xy} of three different $ZrTe_5$ single crystals under high magnetic field at 4.2 K.

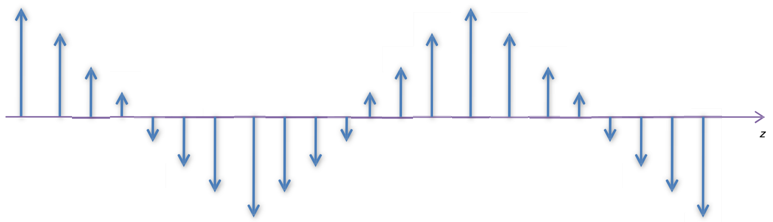

Supplementary Figure 15. The spin density from the *n* = 0 Landau level.

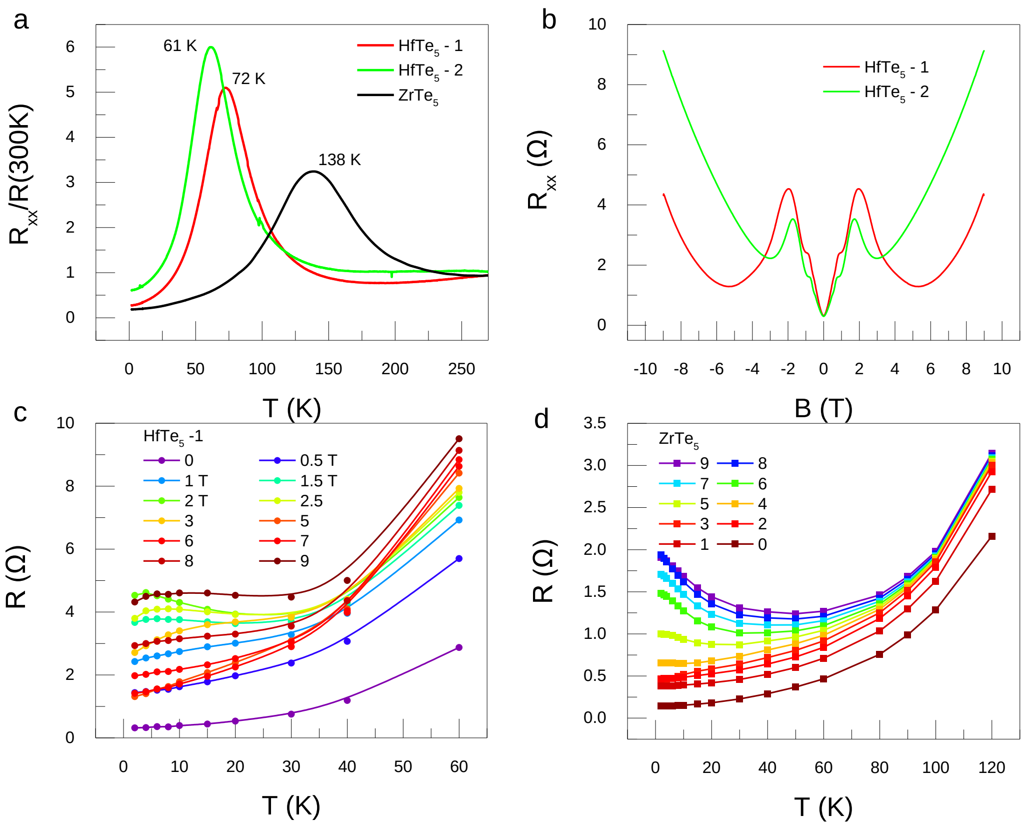

Supplementary Figure 16. Transport properties of $ZrTe_5$ and $HfTe_5$. a, Temperature-dependent resistance of $ZrTe_5$ and $HfTe_5$. **b,** Magnetoresistance of $HfTe_5$. **c,** Temperature-dependent resistance of $HfTe_5$ under different magnetic fields. **d,** Temperature-dependent resistance of $ZrTe_5$ under different magnetic fields.

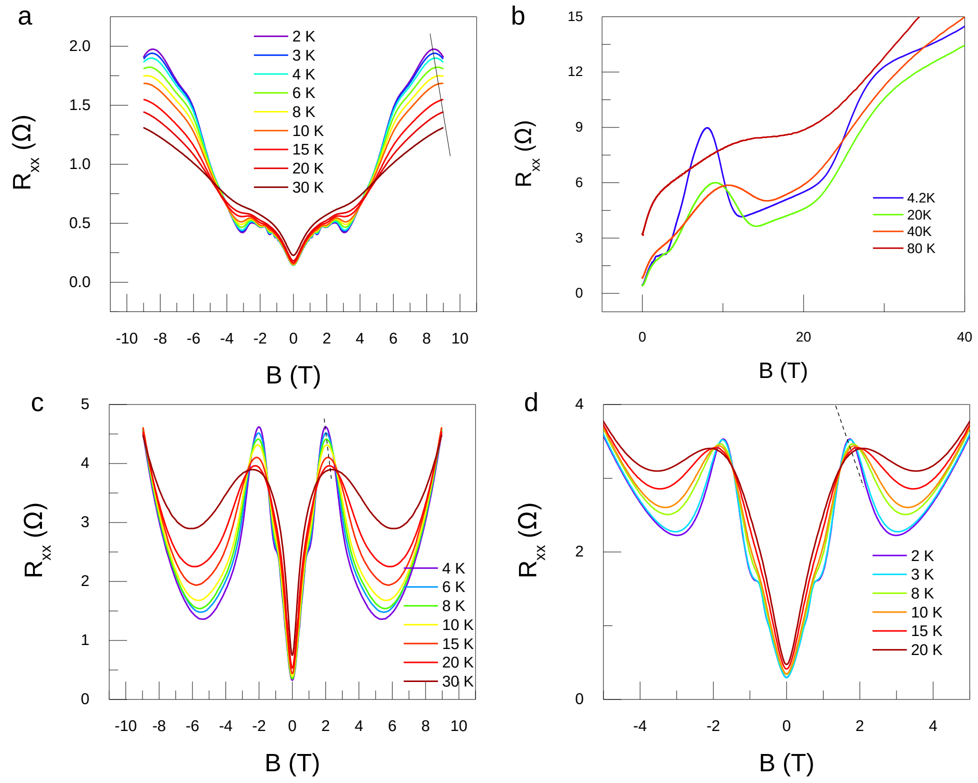

Supplementary Figure 17. Temperature-dependent MR of $ZrTe_5$ and $HfTe_5$. a, Temperature-dependent MR of $ZrTe_5$.**b,** Temperature-dependent MR of $ZrTe_5$ under high magnetic field. **c,** Temperature-dependent MR of $HfTe_5$ -1. **d,** Temperature-dependent MR of $HfTe_5$ -2.

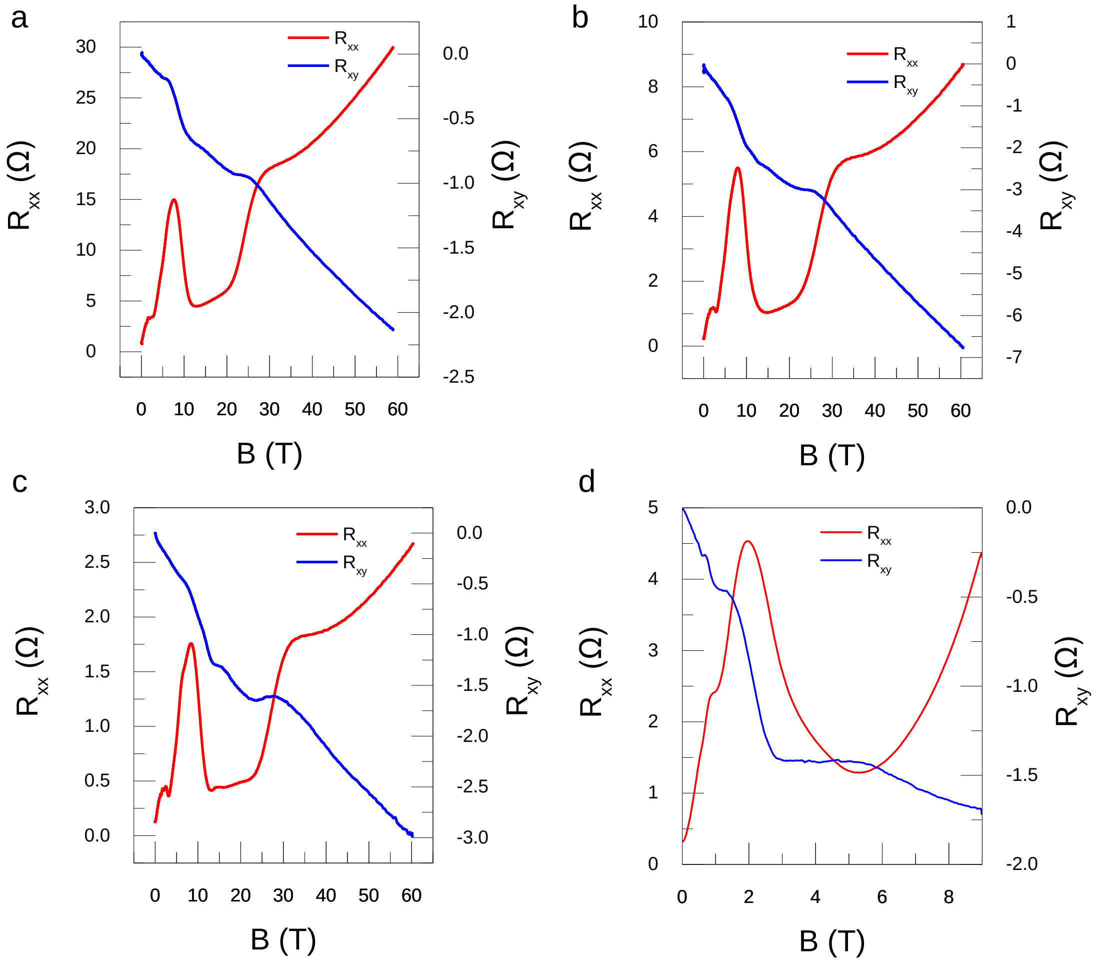

Supplementary Figure 18. Quantum Hall state accompanied with the FISDW state. a-c, the MR and Hall data of three different $ZrTe_5$ samples. **b,** the MR and Hall data of $HfTe_5$.

Supplementary Table 1| Summary of Berry's phase of different Dirac materials

Material	Intercept	Ref.
BiTeI	0.02	2
BiTeCl	~±0.125	3
Ag_2Se	-0.15±0.05	4
$SrMnBi_2$	0.1±0.09	5,6
$CaMnBi_2$	-0.05	7
$SrMnSb_2$	-0.04	8
$EuMnBi_2$	-0.12	9

$YbMnBi_2$	0.21	10

Supplementary Note 1. Band structure calculations

To get an insight into the electronic structure of $ZrTe_5$, we carried out *ab initio* density functional theory calculations. First-principles calculations were performed using the Quantum-Espresso package[11]. Perdew-Burke-Ernzerhof form of the exchange-correlation functional was employed[12]. Plane wave cut-off of 40 Ry was used. Brillouin zone was sampled using an $8 \times 8 \times 4$ Monkhorst-Pack *k*-point mesh for the self-consistent calculations. Fermi surface was plotted over a grid of 5577 *k*-points using XCrySDen[13]. Spin-orbit interactions were included in all computations.

According to our calculations, there is a gap less than 50 meV at the Γ point if we employ the experimental lattice constant in the previous literature[1], which is similar to the recent theoretical calculations[14]. Meanwhile, another study shows that the bulk of $ZrTe_5$ is semimetal with band inversion[15]. However, just 1% deviation from the experimental parameters can close the band gap (Supplementary Figure 1d-e), which is exactly the relaxed lattice constants[14]. Moreover, we found that the value of the band gap is highly tunable by the lattice constants (Supplementary Figure 1c). The evolution of Fermi surface verse Fermi energy is also simulated (Supplementary Figure 2). When the Fermi level is located in the conduction band, there is a highly anisotropic Fermi ellipsoid in the middle of the Brillouin zone, which strongly supports the results that we concluded from the analysis of SdH oscillations in the main text. Besides, in the rest of the Brillouin zone, there are some open Fermi surfaces which may come from other bands. It is also consistent with our Hall analysis in the Supplementary Note 2.

Supplementary Note 2. Multi-carrier transport

The MR and Hall resistance show exotic temperature-dependent behavior. Three types of MR can be classified: in the high temperature regime before the *p-n* transition

(160~300 K), the MR exhibits a classical parabolic relation with magnetic field B; in the intermediate regime (40~150 K), the MR displays clearly a saturation and then at lower temperature (<20 K), the quantum effect dominates and quantum oscillations occur (Supplementary Figure 3).

The temperature-dependent MR suggests a multi-carrier transport. We start with the high temperature regime. At 300 K, the MR of $ZrTe_5$ shows a parabolic behavior, and the Hall resistance exhibits a positive slope, indicating a hole-dominated transport (Supplementary Figure 4). As the temperature decreases, the MR ratio increases and it becomes saturated under high magnetic field, meanwhile the linear positive Hall resistance gradually changes to an *S* shape with negative slope at the high-field regime. It suggests that two types of carriers begin to contribute to the transport. Indeed, the violation of the Kohler's rule clearly demonstrates the classical multi-carrier transport. According to the Kohler's rule[16–18],

$$\frac{R_{xx}(B,T)}{R_{xx}(0,T)} = F\left(\frac{B}{R_{xx}(0,T)}\right), \tag{1}$$

the MR at different temperatures can be rescaled by the Kohler plot. If there is one type of carrier with the same scattering time at the Fermi surface everywhere, the temperature-dependent Kohler plot of the MR curve would overlap each other. However, in the high temperature regime (160~300 K), the Kohler's plot at different temperatures falls on different collapsed curves, providing unambiguous evidence of multi-carrier transport (Supplementary Figure 4c). Similar analysis can be applied to the low temperature and the intermediate temperature regime (2~138 K) after the *p-n* transition. The MR shows quantum effect at around 30 K, where the MR saturation behavior tends to be suppressed (Supplementary Figure 5a). At lower temperatures the quantum oscillations emerge and its MR behavior is primarily dominated by quantum transport rather than the previous classical transport. The Hall resistance shows a negative slope when the temperature is lower than 100 K (Supplementary Figure 5b). However, the nonlinear Hall resistance and the violation of the Kohler's rule (Supplementary Figure 5c) demonstrate that at low temperatures there are two types of electrons contributing to the magneto-transport, as discussed below.

To quantitatively analyze the multi-carrier transport, two-carrier transport model is adopted. For better accuracy, the R_{xx} and R_{xy} are converted to conductance G_{xy} by $\mathrm{G_{xy}} = \frac{R_{xy}}{R_{xx}^2+R_{xy}^2}$ and then G_{xy} at different temperatures are fitted to the two-carrier model[18,19]: $\mathrm{G_{xy}} = \sum_i \frac{n_i e \mu_i^2 B}{[1+(\mu_i B)^2]}$. Here n_i and μ_i denote the carrier density and mobility for each carrier, respectively. By preforming the best fit, the carrier density and mobility for different carriers can be extracted (Supplementary Figure 6). At low temperatures, two types of electrons are found, one with a high mobility up to 50,000 $\mathrm{cm^2V^{-1}s^{-1}}$ and the other with a low mobility of 5,000 $\mathrm{cm^2V^{-1}s^{-1}}$ (Supplementary Figure 7b). The former one can be regarded as the origin of the quantum oscillations which hosts the Dirac nature as extensively discussed in the main text. The latter one, however, may come from a parabolic band and does not cause SdH oscillations owing to a large scattering rate or an open orbit[16]. These experimental observations are consistent with our band structure calculations as presented later in the Supplementary Note 1. It should be noticed that both the mobility and carrier density of two types of electrons saturate below 20 K (Supplementary Figure 7a-b). As a result, their conductance ratio ($G=ne\mu$) remains almost a constant and high-mobility electrons dominate the transport below 20 K. As the temperature increases, the low mobility electron gradually dominates the transport at ~40 K (the dashed line in Supplementary Figure 7c). Correspondingly, the MR feature changes from the quantum oscillations to the classical saturated MR as mentioned above (Supplementary Figure 5a).

The temperature-dependent carrier density clearly shows temperature-dependent Fermi level. At low temperatures, the carrier density of the low mobility electrons increases with increasing temperature (Supplementary Figure 7a). At about 80 K, the carrier density drops abruptly showing that the Fermi level starts to leave the bottom of conduction band. Meanwhile, the carrier density of the high mobility electrons has almost dropped to zero and then converts to hole carriers continuously (Supplementary Figure 8b). The experiments suggest that the Fermi energy shifts from conduction band to valence band with increasing temperature, which agrees well with the recent ARPES results[20,21].

Supplementary Note 3. SdH oscillation analysis

Different magnetic field orientation was taken to probe the Fermi surface and topological phase of $ZrTe_5$. With the similar discussions from the main text, the MR of $ZrTe_5$ with magnetic field in a-b plane is shown in Supplementary Figure 9. A highly anisotropic MR is also observed (Supplementary Figure 9a). With the Landau fan diagram, oscillation frequency and phase factor could be extracted (Supplementary Figure 9b-c). Similar to the scenario where B is rotated inside the b-c plane, the frequency deviates from the law of cosines as the magnetic field approaches the a-axis. Meanwhile, the Berry's phase shifts from nontrivial to trivial.

It is worth noting that the positive MR and quantum oscillations are observed with magnetic field parallel to the a-axis, *i.e.*, the direction of the applied current (Supplementary Figure 10a). With this geometry, chiral magnetic effect was observed in previous transport experiments[22]. The absence of negative MR is probably because of the high Fermi level in our crystals, which also leads to detectable Fermi area and visible quantum oscillations (Supplementary Figure 10b). As discussed in the main text, effective mass can be obtained by fitting the temperature-dependent oscillation amplitude to the temperature smearing factor R_T (Supplementary Figure 10d). And the quantum lifetime can be acquired for the Dingle factor $R_D \sim e^{-D}$, where $D = \frac{\pi m^*}{eB\tau}$ and τ is the quantum lifetime. Using $\Delta R \propto e^{-D}\lambda(T)/sinh\lambda(T)$, we can obtain D from the slope of linear fit to $\ln[\Delta RBsinh\lambda(T)]$ as a function of $1/B$ (Supplementary Figure 10c; Supplementary Figure 11c-d). The lifetime is around 0.2 ps for three principle axes, much lower than the transport lifetime which is determined by the Hall mobility. Such a relationship is also observed in the Dirac semimetal Cd_3As_2[23,24]. This is because the small angle scattering dominates in the low-temperature magneto-transport.

The band structure parameters extracted from the SdH oscillations are summarized in Table 1 in the main text.

In the previous reports[25–27], both multi-frequency and single-frequency quantum oscillations have been reported. Recently, several papers concerning the low-field transport of $ZrTe_5$ bulk nanostructures also claimed the single-frequency quantum oscillations[28,29]. These experimental results nicely suggest that in most cases single-frequency quantum oscillations are observable which is the result of Fermi level position. Recently, ARPES data show that the band structure of $ZrTe_5$ highly depends on the temperature and the Fermi energy[21,30,22]. As the temperature decreases below the temperature-anomaly point, Fermi level moves from the valence band into the conduction band. Further reducing the temperature moves the Fermi level deeper into the conduction band. The topology of the Fermi surface can vary with different Fermi energy. It is worth noting that the multi-frequency quantum oscillations were observed on the sample showing resistivity anomaly at 170 K in Ref. [27], which is much higher than that of the single-frequency case. This means that the *p-n* transition occurs at higher temperature with a deeper Fermi level into the conduction band at low temperature. With such a high Fermi energy, it is indeed possible to involve multiple bands to contribute to the quantum oscillations. And in our case, the resistivity anomaly occurs at 138 K, indicating the lower Fermi energy at low temperature. Thus, unlike the previous research in Ref. [27], we observed single-frequency quantum oscillations. In short, the different Fermi levels will result in distinct quantum oscillation behavior.

It worth noting that the intercept γ for the non-trivial state is around 0.14, which is deviated from 0, the value for strictly perfect 2D case. However, it is not contradicted to the quasi-2D feature of $ZrTe_5$. For $ZrTe_5$, because of its weak interlayer interaction[14], it is reasonable to obtain a highly anisotropic Fermi surface, which gives us a property of quasi-two dimensional. And the quasi-2D Dirac feature have been unambiguously demonstrated by several evidence in our manuscript:

(1) Our angular-dependent magneto-transport results suggest that the quasi-2D feature survives up to 70° even under high magnetic field (Fig. 2c and Fig. 4b in the manuscript). The periodicity of the angular quantum oscillations depends on the law of cosines (the inset of Fig. 2c in the manuscript and Supplementary Figure 9c). Within

70°, the Berry's phase remains close to the non-trivial π Berry's phase, suggesting the Dirac fermion with a strong quasi-2D feature.

(2) Another very important evidence is the layered-transport and the quantum Hall effect, as shown in Fig. 2f. Recently, such behavior was also observed in $EuMnBi_2$[9], which was believed to originate from the 2D Dirac fermion. We have carefully read the reference 9. In fact, the gamma for $EuMnBi_2$ in that reference varies from 0.08 to 0.12, closed to 1/8, rather than strictly 0.

(3) The high-field measurements also confirm the quasi-2D feature (Fig. 4b). Under the ultra-high magnetic field, the quasi-2D feature is still robust.

All these results support that we observe a quasi-2D Dirac fermion in a-c plane. However, once we further tilted the magnetic field beyond 70 °, the oscillations behavior significantly deviated from the 2D case. From our experiments, we observe quantum oscillations when the magnetic field is along three principle axes which indicate the 3D Fermi surface topology of our $ZrTe_5$ sample, instead of a perfect 2D Fermi surface. However, this is not contradictory to our claim of $ZrTe_5$ being as a quasi-two-dimensional Dirac material. As the magnetic is rotated towards the *a*-axis or *c*-axis, the Berry's phase turns to be trivial (Fig. 2 and Supplementary Figure 9). The gamma is 0.5 when the magnetic field is along *a*-axis or *c*-axis, suggesting the trivial state. In other words, non-trivial Berry's phase is only observed within 70° where the quasi-2D feature is present. Thus, we would like to clearly state that based on our experiments $ZrTe_5$ has a highly anisotropic 3D Fermi surface topology, but it has quasi-2D Dirac feature in a-c plane as evidenced by the nontrivial Berry phase whereas along the *a*-axis or *c*-axis it turns to a trivial Berry phase, indicating the non-Dirac property.

In terms of the intercept gamma with respect to the dimensionality of the Fermi surface, it is a debatable method to determine the dimensionality by Berry's phase experimentally. Recently such kind of quasi-two dimensional Fermi surface was also reported in other Dirac materials such as BiTeI[2], BiTeCl[3], Ag_2Se[4], TaAs[31], NbAs[32], $SrMnBi_2$[5,6], $CaMnBi_2$[7], $SrMnSb_2$[8], $EuMnBi_2$[9] and $YbMnBi_2$[10]. However, their intercepts of Landau fan diagram vary significantly, rather than strictly 0, as summarized in the Supplementary Table 1. There are substantial interactions that

influence the Berry's phase. The spin-orbit coupling effect, the Zeeman effect, the non-ideal Dirac nature, nested Fermi surface or the broken particle-hole symmetry would generate the deviation of Berry' phase from non-trivial π. The 3D Fermi surface topology rather than strictly perfect 2D Fermi surface of $ZrTe_5$ is possible to cause some deviation of Berry's phase. And the prominent Zeeman effect could also make the gamma deviated from 0. Here we would like to summarize the intercept of the Landau diagram for various materials reported recently.

Therefore, overall $ZrTe_5$ has a highly anisotropic 3D Fermi surface topology, but in a wide range of angle, it shows a 2D Dirac feature. Thus we can describe it as a quasi-two dimensional Dirac material. The observation of a non-zero gamma value is not contradictory to the claim of the quasi-2D Dirac feature as it can be influenced by other factors.

Supplementary Note 4. Zeeman splitting of R_{xx} and R_{xy}

The Zeeman effect here will not affect our previous analysis of SdH oscillations. To be explicit, we start with the general features of the SdH oscillations from our experiments: (1) The Zeeman effect can be observed distinctly at low temperature below 3 K. (2) Above 2 K, only the Zeeman splitting of 2nd Landau levels can be distinguished from the R_{xx} signals. The 3th and higher Landau levels do not exhibit sizable Zeeman splitting. (3) To observe the Zeeman effect clearly, we have to reduce the temperature down to 400 mK.

When we extracted the carrier transport parameters, it was necessary to neglect the influence from the Zeeman effect. We focused on the Landau levels higher than 3rd, where the Zeeman effect was negligible. Also, the temperature is higher than 2 K to reduce the influence from Zeeman effect. Subsequently, the effective mass was extracted by the SdH oscillations in Fig. 2d at 1.72 T, 1.45 T and 1.25 T, respectively. And the Zeeman factor R_s doesn't affect the analysis at this stage, as shown by the eq.(1).

When we analyzed the Zeeman effect, however, we needed to reduce the system temperature to milikelvin range and focus on the lower Landau levels which exhibited a prominent Zeeman effect. For the sake of simplicity, the Zeeman factor $R_S = \cos(\frac{\pi g m^*}{2m_0})$ can be merged into $\cos 2\pi(\frac{S_F}{B}+\gamma)$ in eq. (1), taking the following mathematical process:

$$\begin{aligned}
\Delta R_{xx} &\propto R_T R_D R_S \cos 2\pi(\frac{S_F}{B}+\gamma) \\
&\propto R_T R_D \cos(\frac{\pi g m^*}{2m_0}) \cos 2\pi(\frac{S_F}{B}+\gamma) \\
&\propto R_T R_D [\cos 2\pi[(\frac{S_F}{B}+\gamma)+\frac{\pi g m^*}{2m_0})] + \cos 2\pi[(\frac{S_F}{B}+\gamma)-\frac{\pi g m^*}{2m_0})]] \\
&\propto R_T R_D \{\cos[2\pi(\frac{S_F}{B}+\gamma)+\pi\varphi] + \cos[2\pi(\frac{S_F}{B}+\gamma)-\pi\varphi]\} \\
&\propto R_T R_D [\cos 2\pi(\frac{S_F}{B}+\gamma+\frac{1}{2}\varphi) + \cos 2\pi(\frac{S_F}{B}+\gamma-\frac{1}{2}\varphi)],
\end{aligned}$$

where $\varphi = \frac{g m^*}{2m_0}$. The final equation is shown in eq.(2). So eq.(1) and eq.(2) are equal to each other mathematically.

Supplementary Note 5. Explicit Landau level wavefunctions

According to symmetry considerations[14,33], a low-energy Hamiltonian (without magnetic field) for $ZrTe_5$ is

$$H_0(\mathbf{k}) = \hbar(v_x k_x \tau_x \sigma_z + v_y k_y \tau_y + v_z k_z \tau_x \sigma_x) + m\tau_z, \tag{1}$$

where the Pauli matrices $\tau_{x,y,z}$ refer to certain orbital degrees of freedom, while $\sigma_{x,y,z}$ refers to the electron spin. The x, y, z axes correspond to a, c, b axes of the crystal, respectively.

Suppose that the g-factor of the $\tau_z = \pm 1$ orbital is $g = \bar{g} \pm \delta g$, the Zeeman coupling reads

$$H_{\text{Zeeman}} = -\mu_B(\bar{g} + \delta g \tau_z)\frac{\sigma_z}{2}B \tag{2}$$

Since $ZrTe_5$ is a highly quasi-two-dimensional system, the Landau level formation in the magnetic field is most significant in the *xy* plane, therefore, we first study the Landau level induced by a magnetic field $\mathbf{B} = B\mathbf{e}_z$, using the Landau gauge $\mathbf{A} = (-By, 0, 0)$.

Now we solve the Landau levels for the full Hamiltonian $H(\mathbf{k}) = H_0(\mathbf{k}) + H_{\text{Zeeman}}$. The Landau level problem for magnetic field along z direction has also been solved in Ref. [33], while we shall solve it in a way convenient for our problem. Let us take the standard replacement $\hbar k_i \to -i\hbar\partial_i + eA_i \equiv \hat{p}_i$ for $i = x, y$. These operators satisfy the commutation relation $[\hat{p}_x, \hat{p}_y] = -i\hbar eB$. The Hamiltonian in the presence of magnetic field takes the form of

$$H = \begin{bmatrix} m - E_z - \delta E_z & 0 & v_x\hat{p}_x - iv_y\hat{p}_y & E_k \\ 0 & m + E_z + \delta E_z & E_k & -v_x\hat{p}_x - iv_y\hat{p}_y \\ v_x\hat{p}_x + iv_y\hat{p}_y & E_k & -m - E_z + \delta E_z & 0 \\ E_k & -v_x\hat{p}_x + iv_y\hat{p}_y & 0 & -m + E_z - \delta E_z \end{bmatrix}, \tag{3}$$

in which we have defined several shorthand notations $E_z = \mu_B\bar{g}B/2$, $\delta E_z = \mu_B\delta gB/2$, and $E_k = \hbar v_z k_z = v_z p_z$ (to simplify notations, hereafter we shall denote k_z as *k*). Now we have the commutation relation

$$[v_x\hat{p}_x - iv_y\hat{p}_y, v_x\hat{p}_x + iv_y\hat{p}_y] = 2\hbar v_x v_y eB, \tag{4}$$

therefore, we can define the ladder operators

$$\begin{aligned} b &= (v_x\hat{p}_x - iv_y\hat{p}_y)/E_B, \\ b^\dagger &= (v_x\hat{p}_x + iv_y\hat{p}_y)/E_B, \end{aligned} \tag{5}$$

with $E_B \equiv \sqrt{2\hbar v_x v_y eB}$ (we have supposed that $v_x v_y B > 0$, and the $v_x v_y B < 0$ case is similar; from our data, we have $E_B = \sqrt{2\hbar v_x v_y eB} \approx 19.0\sqrt{B}\text{meV}$), such that

$$[b, b^\dagger] = 1. \tag{6}$$

With this notation, the Hamiltonian becomes

$$H=\begin{bmatrix} m-E_z-\delta E_z & 0 & E_B b & E_k \\ 0 & m+E_z+\delta E_z & E_k & -E_B b^\dagger \\ E_B b^\dagger & E_k & -m-E_z+\delta E_z & 0 \\ E_k & -E_B b & 0 & -m+E_z-\delta E_z \end{bmatrix}, \qquad (7)$$

Let us denote the eigenfunction of $b^\dagger b$ with eigenvalue n as ϕ_n. The Landau levels can be solved as follows. We take the ansatz $\psi=\begin{pmatrix} a_1\phi_{n-1} \\ a_2\phi_n \\ a_3\phi_n, \\ a_4\phi_{n-1} \end{pmatrix}$, thus the Schrodinger equation becomes

$$\begin{bmatrix} m-E_z-\delta E_z & 0 & E_B\sqrt{n} & E_k \\ 0 & m+E_z+\delta E_z & E_k & -E_B\sqrt{n} \\ E_B\sqrt{n} & E_k & -m-E_z+\delta E_z & 0 \\ E_k & -E_B\sqrt{n} & 0 & -m+E_z-\delta E_z \end{bmatrix}\begin{pmatrix} a_1 \\ a_2 \\ a_3 \\ a_4 \end{pmatrix}=E\begin{pmatrix} a_1 \\ a_2 \\ a_3 \\ a_4 \end{pmatrix}. \qquad (8)$$

The m term is known to be tiny, and the Zeeman splitting can be accounted for by the E_z term, thus it is reasonable to discard the m and δE_z terms, which enables simpler analytic solutions. The $n=0$ Landau levels have two instead of four energy eigenvalues

$$E_{0,s}=s\sqrt{E_k^2+E_B^2}, \qquad (9)$$

where $s=\pm1$. For the $s=+1$ mode, the wavefunction is

$$\psi_{n=0,s=+1}=\begin{pmatrix} 0 \\ \cos(\theta_k/2) \\ \sin(\theta_k/2) \\ 0 \end{pmatrix}\phi_0, \qquad (10)$$

while for the $s=-1$ mode, the wavefunction is

$$\psi_{n=0,s=-1}=\begin{pmatrix} 0 \\ \sin(\theta_k/2) \\ -\cos(\theta_k/2) \\ 0 \end{pmatrix}\phi_0, \qquad (11)$$

where θ_k satisfies $\tan\theta_k=E_k/E_z$.

The Landau levels energy eigenvalues for $n \neq 0$ are

$$E_{n,\alpha,s} = \alpha\sqrt{E_k^2 + (E_B\sqrt{n} + sE_z)^2}, \qquad (12)$$

with $\alpha = \pm 1$ and $s = \pm 1$. By explicitly solving the equations, we can also see that $s = \pm 1$ corresponds to $\tau_x = \mp 1$. The eigenfunctions of the $s = +1$ modes are

$$\psi_{n,s=+1,\alpha=+1} = \begin{pmatrix} \sin(\theta_{k,n}/2)\phi_{n-1} \\ -\cos(\theta_{k,n}/2)\phi_n \\ -\sin(\theta_{k,n}/2)\phi_n \\ \cos(\theta_{k,n}/2)\phi_{n-1} \end{pmatrix}; \psi_{n,s=+1,\alpha=-1} = \begin{pmatrix} \cos(\theta_{k,n}/2)\phi_{n-1} \\ \sin(\theta_{k,n}/2)\phi_n \\ -\cos(\theta_{k,n}/2)\phi_n \\ -\sin(\theta_{k,n}/2)\phi_{n-1} \end{pmatrix}, \qquad (13)$$

where $\theta_{k,n}$ is defined by

$$\tan\theta_{k,n} = E_k / (E_B\sqrt{n} + E_z). \qquad (14)$$

Similarly, the eigenfunctions for the $s = -1$ modes are

$$\psi_{n,s=-1,\alpha=-1} = \begin{pmatrix} \cos(\theta_{k,n}/2)\phi_{n-1} \\ \sin(\theta_{k,n}/2)\phi_n \\ \cos(\theta_{k,n}/2)\phi_n \\ \sin(\theta_{k,n}/2)\phi_{n-1} \end{pmatrix}; \psi_{n,s=-1,\alpha=+1} = \begin{pmatrix} \sin(\theta_{k,n}/2)\phi_{n-1} \\ -\cos(\theta_{k,n}/2)\phi_n \\ \sin(\theta_{k,n}/2)\phi_n \\ -\cos(\theta_{k,n}/2)\phi_{n-1} \end{pmatrix}. \qquad (15)$$

The explicit Landau level wavefunctions will be useful in determining the form of density wave to be discussed below.

Now let us calculate the Landau levels for magnetic field along the *x* direction. To simplify the calculation, it is convenient to rotate the spin as

$$\sigma_x \to \sigma_z, \sigma_z \to -\sigma_x, \qquad (16)$$

such that the Hamiltonian takes the form of

$$H = \hbar(-v_x k_x \tau_x \sigma_x + v_y k_y \tau_y + v_z k_z \tau_x \sigma_z) + m\tau_z - E_z\sigma_z. \qquad (17)$$

Note that we have included the Zeeman term, which takes the form of $-E_z\sigma_x$ in the original basis, and $-E_z\sigma_z$ in the spin-rotated basis.

Let us follow the notations of the previous section. The momentum operators satisfy the commutation relation $[\hat{p}_y, \hat{p}_z] = -i\hbar eB$. The Hamiltonian in the presence of magnetic field takes the form of

$$H = \begin{bmatrix} m - E_z & 0 & v_z \hat{p}_z - i v_y \hat{p}_y & -E_k \\ 0 & m + E_z & -E_k & -v_z \hat{p}_z - i v_y \hat{p}_y \\ v_z \hat{p}_z + i v_y \hat{p}_y & -E_k & -m - E_z & 0 \\ -E_k & -v_z \hat{p}_z + i v_y \hat{p}_y & 0 & -m + E_z \end{bmatrix}, \tag{18}$$

in which we have defined several shorthand notations $E_z = \mu_B \bar{g} B / 2$, and $E_k = \hbar v_x k_x = v_x p_x$ (to simplify notations, hereafter in this section we shall denote k_x as k). Now we have the commutation relation

$$[v_z \hat{p}_z - i v_y \hat{p}_y, v_z \hat{p}_z + i v_y \hat{p}_y] = -2\hbar v_x v_y eB, \tag{19}$$

therefore, we can define the ladder operators

$$\begin{aligned} b &= (v_z \hat{p}_z + i v_y \hat{p}_y) / E_B, \\ b^\dagger &= (v_z \hat{p}_z - i v_y \hat{p}_y) / E_B, \end{aligned} \tag{20}$$

with $E_B \equiv \sqrt{2\hbar v_y v_z eB}$ (we have supposed that $v_y v_z B > 0$, and the $v_y v_z B < 0$ case is similar), such that

$$[b, b^\dagger] = 1. \tag{21}$$

With this notation, the Hamiltonian becomes

$$H = \begin{bmatrix} m - E_z & 0 & E_B b^\dagger & -E_k \\ 0 & m + E_z & -E_k & -E_B b \\ E_B b & -E_k & -E_z & 0 \\ -E_k & -E_B b^\dagger & 0 & -m + E_z \end{bmatrix}. \tag{22}$$

Let us denote the eigenfunction of $b^\dagger b$ with eigenvalue n as ϕ_n . The Landau levels can be solved as follows. We take the ansatz $\psi = \begin{pmatrix} a_1 \phi_n \\ a_2 \phi_{n-1} \\ a_3 \phi_{n-1}, \\ a_4 \phi_n \end{pmatrix}$, thus the Schrodinger equation becomes

$$\begin{bmatrix} m - E_z & 0 & E_B \sqrt{n} & -E_k \\ 0 & m + E_z & -E_k & -E_B \sqrt{n} \\ E_B \sqrt{n} & -E_k & -m - E_z & 0 \\ -E_k & -E_B \sqrt{n} & 0 & -m + E_z \end{bmatrix} \begin{pmatrix} a_1 \\ a_2 \\ a_3 \\ a_4 \end{pmatrix} = E \begin{pmatrix} a_1 \\ a_2 \\ a_3 \\ a_4 \end{pmatrix}. \tag{23}$$

Let us omit the tiny m term. The n = 0 Landau levels have two instead of four energy

eigenvalues

$$E_{0,s} = s\sqrt{E_k^2 + E_B^2}, \tag{24}$$

where $s = \pm 1$. We shall not be concerned with the wavefunctions for magnetic field along *x* direction.

The Landau levels energy eigenvalues for $n \neq 0$ are

$$E_{n,\alpha,s} = \alpha\sqrt{E_k^2 + (E_B\sqrt{n} + sE_z)^2}, \tag{25}$$

with $\alpha = \pm 1$ and $s = \pm 1$. By explicitly solving the equations, we can also see that $s = \pm 1$ corresponds to $\tau_x = \mp 1$. From the energy eigenvalues of the Landau levels, we can see that there is Zeeman splitting, which is observable in the transport experiment.

Finally let us calculate the Landau levels for magnetic field along the *y* direction. To simplify the calculation, it is convenient to rotate the spin as

$$\sigma_y \to \sigma_z, \sigma_z \to -\sigma_y, \tag{26}$$

such that the Hamiltonian takes the form of

$$H = \hbar(-v_x k_x \tau_x \sigma_y + v_y p_y \tau_y + v_z k_z \tau_x \sigma_x) + m\tau_z - E_z \sigma_z. \tag{27}$$

Note that we have included the Zeeman term, which takes the form of $-E_z\sigma_y$ in the original basis, and $-E_z\sigma_z$ in the spin-rotated basis.

Let us follow the notations of the previous section. The momentum operators satisfy the commutation relation $[\hat{p}_z, \hat{p}_x] = -i\hbar eB$. The Hamiltonian in the presence of magnetic field takes the form of

$$H = \begin{bmatrix} m - E_z & 0 & -iE_k & iv_x p_x + v_z p_z \\ 0 & m + E_z & -iv_x p_x + v_z p_z & -iE_k \\ iE_k & iv_x p_x + v_z p_z & -m - E_z & 0 \\ -iv_x p_x + v_z p_z & iE_k & 0 & -m + E_z \end{bmatrix}, \tag{28}$$

in which we have defined several shorthand notations $E_z = \mu_B \bar{g} B/2$, and $E_k = \hbar v_y k_y = v_y p_y$ (to simplify notations, hereafter in this section we shall denote k_y as *k*). Now we have the commutation relation

$$[v_z\hat{p}_z - iv_x\hat{p}_x, v_z\hat{p}_z + iv_x\hat{p}_x] = 2\hbar v_x v_y eB, \tag{29}$$

therefore, we can define the ladder operators

$$\begin{aligned} b &= (v_z\hat{p}_z - iv_x\hat{p}_x)/E_B, \\ b^\dagger &= (v_z\hat{p}_z - iv_x\hat{p}_x)/E_B, \end{aligned} \tag{30}$$

with $E_B \equiv \sqrt{2\hbar v_x v_z eB}$ (we have supposed that $v_x v_z B > 0$, and the $v_x v_z B < 0$ case is similar), such that

$$[b, b^\dagger] = 1. \tag{31}$$

With this notation, the Hamiltonian becomes

$$H = \begin{bmatrix} m - E_z & 0 & -iE_k & E_B b^\dagger \\ 0 & m + E_z & E_B b & -iE_k \\ iE_k & E_B b^\dagger & -m - E_z & 0 \\ E_B b & iE_k & 0 & -m + E_z \end{bmatrix}. \tag{32}$$

Let us denote the eigenfunction of $b^\dagger b$ with eigenvalue n as ϕ_n. The Landau levels can be solved as follows. We take the ansatz $\psi = \begin{pmatrix} a_1\phi_n \\ a_2\phi_{n-1} \\ a_3\phi_n, \\ a_4\phi_{n-1} \end{pmatrix}$, thus the Schrodinger equation becomes

$$\begin{bmatrix} m - E_z & 0 & -iE_k & E_B\sqrt{n} \\ 0 & m + E_z & E_B\sqrt{n} & -iE_k \\ iE_k & E_B\sqrt{n} & -m - E_z & 0 \\ E_B\sqrt{n} & iE_k & 0 & -m + E_z \end{bmatrix} \begin{pmatrix} a_1 \\ a_2 \\ a_3 \\ a_4 \end{pmatrix} = E \begin{pmatrix} a_1 \\ a_2 \\ a_3 \\ a_4 \end{pmatrix}. \tag{33}$$

Let us omit the tiny m term. The n = 0 Landau levels have two instead of four energy eigenvalues

$$E_{0,s} = sE_k - E_z, \tag{34}$$

where $s = \pm 1$ are identical to the τ_y eigenvalues. The wavefunctions of the n = 0 Landau levels have $\sigma_z = +1$. We shall not be concerned with the explicit form of the wavefunctions for magnetic field along y direction.

The Landau levels energy eigenvalues for $n \neq 0$ are

$$E_{n,\alpha,s} = \alpha\sqrt{nE_B^2 + (E_k + sE_z)^2}, \tag{35}$$

with $\alpha = \pm 1$ and $s = \pm 1$. From this spectrum we can see that the presence of Zeeman term E_z merely shifts the spectrum, without causing Zeeman splitting in the SdH oscillations. This is in sharp contrast to the cases of magnetic field along x or z direction, for which we have the combination "$\sqrt{n}E_B + sE_z$" instead of "$E_k + sE_z$".

Supplementary Note 6. High-field measurements

The high-field measurements were carried out under the pulsed magnetic field up to 60 T. Several samples have been measured, as shown in Supplementary Figure 14. Similar transport behavior has been observed as described in the main text. When the magnetic field is applied along a-axis, Zeeman splitting is also observed (Supplementary Figure 13). And the g-factor is estimated to be 3.19 by the same method as described in the main text.

As the $ZrTe_5$ single-crystals have a long and narrow shape, it is challenging to prepare Hall electrodes for the pulsed high magnetic field measurements. To attain the Hall signals, we have made extensive efforts to measure samples with Hall electrodes. Consistent with the layered-transport in the low field regime as discussed in the main text, the Hall resistance shows large plateaus (Supplementary Figure 14), indicating the bulk quantum Hall effect[9]. It is well known that the quantum Hall effect is accompanied by a cascade of field-induced density wave state in a mount of quasi-1D organic conductors[34]. As the field changes, the system experiences a cascade of phase transitions between the field-induced density wave states and the different index of quantum Hall states. As a result, a series of quantum Hall plateaus will be separated by phase transitions. In our scenario, we observed the distinct plateaus within the reentrant regime between ~12 T and ~22 T, which is also the regime between two field-induced density wave states as indicated by the abrupt resistivity increase. So the high-field Hall data also support our explanations of field-induced density wave in $ZrTe_5$.

It worth noting that in the previous published paper, they observed negative MR when the magnetic field is along the current direction[22]. The key difference of these two cases lies in the dissimilar Fermi levels of $ZrTe_5$ crystals resulting from two different growth approaches. In Ref. [22], they observe the chiral anomaly from the $ZrTe_5$ crystal that has a temperature anomaly peak at around 60 K in the *R*-*T* curve (Supplementary Figure 13c). This clearly indicates that their sample has a very low Fermi energy at low temperatures. Therefore, a large magnetoresistance is claimed without the presence of any quantum oscillations when the magnetic field is applied perpendicularly to the sample surface. With such a low Fermi energy, they could further observe the chiral anomaly as suggested by the negative magnetoresistance. However, in our case, the $ZrTe_5$ crystal shows temperature anomaly at 138 K. Owing to a relatively high Fermi energy with a tiny Fermi ellipsoid, we can observe clear quantum oscillations with positive magnetoresistance (MR) instead of negative one.

Another important factor is the zero-field resistivity. As mentioned in Ref. [22], it is easier to observe the chiral magnetic effect in materials that have a relatively large zero-field resistivity. In contrast, because of the high Fermi level, our sample has a relatively large residual resistance ratio (RRR). So it is difficult to observe the negative longitudinal MR at low temperature. However, as the temperature increases, the zero field resistivity will increase. Therefore, at higher temperatures, we are able to observe the enhancement of the negative longitudinal MR (Supplementary Figure 13d).

Supplementary Note 7. Density wave transitions inferred from the data and the explicit form of density waves in $ZrTe_5$

Now we analyze the data of high field transport. There is a sharp peak around B = 8 T and a sharp shoulder-like behavior at B = 30 T. We interpret them as possible density wave transitions in the n = 1 and n = 0 Landau levels, respectively.

First let us discuss the possible origin of the large peak of R_{xx} around B = 8 T. According to extrapolation of the locations of peaks and valleys (for $n \geq 2$) in SdH

oscillation curve, the nominal $n = 1$ peak and valley, which is overwhelmed by the large peak around $B = 8$ T, is around 5.6 T and 13.6 T, respectively, therefore, the 8T peak is likely due to an instability in the $n = 1$ Landau level. A density wave formation within the $n = 1$ Landau level can gap out the carriers in the two $n = 1$ Landau levels, leaving a single $n = 0$ Landau level gapless, thus significantly enhancing the resistivity. We believe that this is the most possible explanation of the 8T peak.

This interpretation is supported by the resistivity data in stronger field. The resistivity drops to a much lower value around $B = 14$ T, which is an indication of the reentrant phenomenon[35]. In fact, if we adopt the crude BCS-type estimation, the density wave transition temperature is

$$k_B T_c = 1.14(E_F - E_B)\exp(-\frac{1}{N(0)V}), \tag{36}$$

where $N(0)$ is the density of states at the Fermi level, while V is an interaction parameter. As we increase B, both k_F and E_F decrease. When E_F decreases to E_B, T_c is suppressed to 0, namely, the density wave in the $n = 1$ Landau level is destroyed as we increase B. This is perfectly consistent with our experimental data. This is analogous to the reentrant transition observed in graphite[35].

Further increasing B enhances the density of states in the $n = 0$ Landau level, thus enhances the instability towards density wave transition in the $n = 0$ Landau level. An illustration of the Landau level and the location of Fermi level in this regime is given in Fig. 4d. Experimentally, a sharp shoulder-like increasing in R_{xx} at $B \approx 30T$ is found. This is likely due to a density wave transition coming from the nesting in the $n = 0$ Landau level.

The possible density waves exhibit interesting features in the Dirac and Weyl semimetals[36]. Following the theoretical formalism in Ref. 36, we now calculate the forms of density wave in $ZrTe_5$.

First we study the simpler one of the two types of density waves, namely, the one from the $n = 0$ Landau level. The z-component of the spin density is

$$\langle \sigma_z \rangle \propto \exp(2ik_F z - i\alpha_0)\langle \psi_{n=0,s=+1,k=-k_F} | \sigma_z | \psi_{n=0,s=+1,k=k_F} \rangle + h.c. \tag{37}$$

where α_0 is a constant phase angle, and the Landau level wavefunctions are

$$\psi_{n=0,s=+1,k=k_F} = \begin{pmatrix} 0 \\ \cos(\theta_{k_F}/2) \\ \sin(\theta_{k_F}/2) \\ 0 \end{pmatrix} \phi_0, \psi_{n=0,s=+1,k=-k_F} = \begin{pmatrix} 0 \\ \cos(\theta_{k_F}/2) \\ -\sin(\theta_{k_F}/2) \\ 0 \end{pmatrix} \phi_0, \tag{38}$$

therefore, we have

$$\langle \sigma_z \rangle = m_0 \cos(2k_F z - \alpha_0), \tag{39}$$

where the constant m_0 can be taken as the order parameter of the density wave. An illustration of this spin density wave is given in Supplementary Figure 15.

Similarly, the charge density wave is

$$\langle \rho \rangle \propto \exp(2ik_F z - i\alpha_0)\langle \psi_{n=0,s=+1,k=-k_F} | \psi_{n=0,s=+1,k=k_F} \rangle + h.c., \tag{40}$$

or more explicitly,

$$\begin{aligned} \langle \rho \rangle &= m_0 \cos(2k_F z - \alpha_0)[\cos^2(\theta_{k_F}/2) - \sin^2(\theta_{k_F}/2)] \\ &= m_0 \frac{E_z}{\sqrt{E_z^2 + E_k^2}} \cos(2k_F z - \alpha_0). \end{aligned} \tag{41}$$

In our material, E_z is generally much smaller than E_k, thus $\langle \rho \rangle << \langle \sigma_z \rangle$, in other words, we have the spin instead of charge density wave[36].

We have also calculated the forms of density waves from the transition in the n = 1 Landau levels, and found them to be similar to that from the n = 0 Landau levels (Certainly, the wavevector $2k_F$ is different for them). For the s = +1 channel, the relevant wavefunctions are

$$\psi_{n,s=+1,\alpha=+1,k=k_F} = \begin{pmatrix} \sin(\theta_{k_F,n}/2)\phi_{n-1} \\ -\cos(\theta_{k_F,n}/2)\phi_n \\ -\sin(\theta_{k_F,n}/2)\phi_n \\ \cos(\theta_{k_F,n}/2)\phi_{n-1} \end{pmatrix}; \psi_{n,s=+1,\alpha=+1,k=-k_F} = \begin{pmatrix} -\sin(\theta_{k_F,n}/2)\phi_{n-1} \\ -\cos(\theta_{k_F,n}/2)\phi_n \\ \sin(\theta_{k_F,n}/2)\phi_n \\ \cos(\theta_{k_F,n}/2)\phi_{n-1}. \end{pmatrix}. \tag{42}$$

The density wave again takes the form of $\langle \sigma_z \rangle \sim \cos(2k_F z - \alpha_0)$ (the values of k_F and α_0 are different from that of the n = 0 Landau level). For the s = -1 channel, the resultant density wave takes the similar form, with a k_F only slightly different.

Supplementary Note 8. Evidences for density wave behavior

To confirm the density wave behavior, several important evidences are provided as follows.

First, let us carefully examine the transport data shown in Supplementary Figure 16. We found that the properties of $HfTe_5$ are similar to that of $ZrTe_5$, and thus it is reasonable to observe density wave transitions in $HfTe_5$. In general, $HfTe_5$ has a much smaller Fermi surface than $ZrTe_5$, as shown in Supplementary Figure 16. Two $HfTe_5$ samples have been measured with the anomaly peaks located at 72 K and 61 K, respectively (Supplementary Figure 16a). The analysis of SdH oscillations suggests that the Fermi surface of $HfTe_5$ in the *a*-*c* plane is 1.5 T, and exhibits the nontrivial Berry's phase as indicated by the intercept of ~0.15 extracted from the Landau fan diagram. The resistance peak of $HfTe_5$ at 2 T is the result of FISDW (Supplementary Figure 16b), which is similar to the peak at 8 T for $ZrTe_5$. Several details are worth noting. The anomaly temperature of $HfTe_5$ is much lower than that of the $ZrTe_5$, which is 138 K in our manuscript. This suggests a reduced carrier density in $HfTe_5$, and the analysis of the SdH oscillations gives the Fermi surface of 1.5 T, which is almost one third of the $ZrTe_5$. As a result, the FISDW of the two $HfTe_5$ samples can be observed at lower fields than $ZrTe_5$, which are 1.9 T and 1.6 T, respectively. With such a lower carrier density, the interaction is thus reduced, leading to a smaller amplitude of the spin density wave (Supplementary Figure 16b). It is obvious that the amplitude of FISDW in $HfTe_5$ is much smaller than that in $ZrTe_5$. And between these two $HfTe_5$ samples, the $HfTe_5$ -1 sample has a larger amplitude than $HfTe_5$ -2 (sample No.1 has a higher anomaly temperature and a higher field for density wave transition). This phenomenon supports our conclusion of interaction-induced density wave transition.

Another important evidence is the temperature-dependent resistance (*R*-*T*) under magnetic field. As shown in Supplementary Figure 16c, the *R*-*T* curves show a metallic behavior with small magnetic field less than 1 T. However, with larger magnetic fields, the FISDW transition takes place and the *R*-*T* curves exhibit insulating behavior (B=1.5 T and 2 T). Later, the *R*-*T* curves exhibit the metallic behavior again with increasing

magnetic field (B>3 T). The insulating behavior occurs between 1.5 T and 2 T, where is the regime of anomalous MR peak induced by the FISDW (Supplementary Figure 16b). And after passing this regime, the system has a reentry into the metallic state, thus the *R*-*T* curves shows metallic behavior. Such reentrance behavior is also observed in other field-induced density wave systems such as graphite[37] and organic conductors[34,38]. And for $ZrTe_5$, a similar evolution is also observed. The *R*-*T* curves show metallic behavior with magnetic field less than 3 T, and the insulting state with magnetic larger than 5 T. The evolution of *R*-*T* curves under magnetic field demonstrates the process of metal-to-FISDW transition and the reentrance from FISDW to metal. And the insulating behavior of *R*-*T* also suggests the gap opening, which is consistent with the conclusion of FISDW.

The temperature-dependent MR can also support our conclusion of FISDW (Supplementary Figure 17). The amplitude reduces a lot as increasing the temperature. With higher temperature, the peak position of the FISDW shifts towards higher field, which can be observed in both $ZrTe_5$ and $HfTe_5$. For SdH oscillations, the peak positions for Landau levels should not shift as the temperature changes. Such sensitive temperature-dependent behavior is another proof for the field-induced density wave[37,39].

From the Hall resistance we can find the evidence for the FISDW. Consistent with the layered-transport in the low field regime as discussed in the manuscript, the Hall resistance shows large plateaus (Supplementary Figure 18), indicating the bulk quantum Hall effect[34]. It is well known that the quantum Hall effect is accompanied by a cascade of field-induced density wave state in a mount of organic Bechgaard salts[40–42]. As the field changes, the system experiences a cascade of phase transitions between the field-induced density wave states and the different index of quantum Hall states. As a result, a series of quantum Hall plateaus will be separated by phase transitions. In our scenario, we observed the distinct plateaus within the reentrant regime between ~12 T and ~22 T, which is also the regime between two field-induced density wave states as indicated by the abrupt resistivity increase. So the high-field Hall data also support our

explanations of field-induced density wave in $ZrTe_5$. And the Hall data of $HfTe_5$ is much more remarkable to witness such quantum Hall state accompanied by the FISDW transition.

To summarize, several evidences can support our conclusion of FISDW including 1) the increase of resistance under high magnetic field, whose amplitude is far beyond the level of quantum oscillations; 2) the evolution of *R*-*T* curves under magnetic field. In the FISDW regime the *R*-*T* shows insulating behavior; 3) sensitive temperature dependence of the peak position caused by FISDW; 4) quantum Hall states accompanied with a cascade of FISDW states.

Supplementary References

1. Fjellvåg, H. & Kjekshus, A. Structural properties of $ZrTe_5$ and $HfTe_5$ as seen by powder diffraction. *Solid State Commun.* **60,** 91–93 (1986).
2. Murakawa, H. *et al.* Detection of Berry's phase in a Bulk Rashba semiconductor. *Science* **342,** 1490–1493 (2013).
3. Xiang, F.-X., Wang, X.-L., Veldhorst, M., Dou, S.-X. & Fuhrer, M. S. Observation of topological transition of Fermi surface from a spindle torus to a torus in bulk Rashba spin-split BiTeCl. *Phys. Rev. B* **92,** 035123 (2015).
4. Zhang, C. *et al.* Ultraquantum magnetoresistance in single-crystalline β-Ag_2Se. Preprint at http://arxiv.org/abs/1502.02324 (2015).
5. Park, J. *et al.* Anisotropic Dirac Fermions in a Bi Square Net of $SrMnBi_2$. *Phys. Rev. Lett.* **107,** 126402 (2011).
6. Wang, K., Graf, D., Lei, H., Tozer, S. W. & Petrovic, C. Quantum transport of two-dimensional Dirac fermions in $SrMnBi_2$. *Phys. Rev. B* **84,** 220401 (2011).
7. Wang, K. *et al.* Two-dimensional Dirac fermions and quantum magnetoresistance in $CaMnBi_2$. *Phys. Rev. B* **85,** 041101 (2012).
8. Liu, J. Y. *et al.* Discovery of a topological semimetal phase coexisting with ferromagnetic behavior in $Sr_{1-y}MnSb_2$ (y~0.08). Preprint at http://arxiv.org/abs/1507.07978 (2015).
9. Masuda, H. *et al.* Quantum Hall effect in a bulk antiferromagnet $EuMnBi_2$ with magnetically confined two-dimensional Dirac fermions. *Sci. Adv.* **2,** e1501117 (2016).
10. Wang, A. *et al.* Two-dimensional Dirac fermions in $YbMnBi_2$ antiferromagnet. Preprint at http://arxiv.org/abs/1604.01009 (2016).
11. Giannozzi, P. *et al.* QUANTUM ESPRESSO: a modular and open-source software project for quantum simulations of materials. *J. Phys. Condens. Matter* **21,** 395502 (2009).
12. Perdew, J. P., Burke, K. & Ernzerhof, M. Generalized Gradient Approximation Made Simple. *Phys. Rev. Lett.* **77,** 3865–3868 (1996).
13. Kokalj, A. Computer graphics and graphical user interfaces as tools in simulations of matter at

the atomic scale. *Comput. Mater. Sci.* **28,** 155–168 (2003).
14. Weng, H., Dai, X. & Fang, Z. Transition-Metal Pentatelluride $ZrTe_5$ and $HfTe_5$: A Paradigm for Large-Gap Quantum Spin Hall Insulators. *Phys. Rev. X* **4,** 011002 (2014).
15. Whangbo, M., DiSalvo, F. & Fleming, R. Electronic structure of $ZrTe_5$. *Phys. Rev. B* **26,** 687–689 (1982).
16. Pippard, A. B. *Magnetoresistance in metals* (Cambridge Univ. Press, 1989).
17. Husmann, A. *et al.* Megagauss sensors. *Nature* **417,** 421–424 (2002).
18. Ishiwata, S. *et al.* Extremely high electron mobility in a phonon-glass semimetal. *Nat Mater* **12,** 512–517 (2013).
19. Qu, D. X., Hor, Y. S., Xiong, J., Cava, R. J. & Ong, N. P. Quantum oscillations and hall anomaly of surface states in the topological insulator Bi_2Te_3. *Science* **329,** 821–824 (2010).
20. McIlroy, D. N. *et al.* Observation of a semimetal–semiconductor phase transition in the intermetallic $ZrTe_5$. *J. Phys. Condens. Matter* **16,** L359–L365 (2004).
21. Manzoni, G. *et al.* Ultrafast Optical Control of the Electronic Properties of $ZrTe_5$. *Phys. Rev. Lett.* **115,** 207402 (2015).
22. Li, Q. *et al.* Chiral magnetic effect in $ZrTe_5$. *Nat. Phys.* **12,** 550–554 (2016).
23. Liang, T. *et al.* Ultrahigh mobility and giant magnetoresistance in the Dirac semimetal Cd_3As_2. *Nat Mater* **14,** 280–284 (2015).
24. Narayanan, A. *et al.* Linear Magnetoresistance Caused by Mobility Fluctuations in n-Doped Cd_3As_2. *Phys. Rev. Lett.* **114,** 117201 (2015).
25. Izumi, M. *et al.* Shubnikov-de Haas oscillations and Fermi surfaces in transition-metal pentatellurides $ZrTe_5$ and $HfTe_5$. *J. Phys. C Solid State Phys.* **20,** 3691 (1987).
26. Yoshizaki, R., Izumi, M., Harada, S., Uchinokura, K. & Matsuura, E. Shubnikov-de Haas oscillations in transition-metal pentatelluride. *Phys. BC* **117-118,** 605–607 (1983).
27. Kamm, G., Gillespie, D., Ehrlich, A., Wieting, T. & Levy, F. Fermi surface, effective masses, and Dingle temperatures of $ZrTe_5$ as derived from the Shubnikov–de Haas effect. *Phys. Rev. B* **31,** 7617–7623 (1985).
28. Zheng, G. *et al.* Transport evidence for the three-dimensional Dirac semimetal phase in $ZrTe_5$. *Phys. Rev. B* **93,** 115414 (2016).
29. Yu, W. *et al.* Quantum Oscillations at Integer and Fractional Landau Level Indices in Dirac Semimetal $ZrTe_5$. Preprint at http://arxiv.org/abs/1602.06824 (2016).
30. Zhang, Y. *et al.* Electronic Evidence of Temperature-Induced Lifshitz Transition and Topological Nature in $ZrTe_5$. Preprint at http://arxiv.org/abs/1602.03576 (2016).
31. Zhang, C. *et al.* Tantalum Monoarsenide: an Exotic Compensated Semimetal. Preprint at http://arxiv.org/abs/1502.00251 (2015).
32. Luo, Y. *et al.* A novel electron-hole compensation effect in NbAs. Preprint at http://arxiv.org/abs/1506.01751 (2015).
33. Chen, R. Y. *et al.* Magnetoinfrared Spectroscopy of Landau Levels and Zeeman Splitting of Three-Dimensional Massless Dirac Fermions in $ZrTe_5$. *Phys. Rev. Lett.* **115,** 176404 (2015).
34. *The physics of organic superconductors and conductors*. (Springer, 2008).
35. Yaguchi, H. & Singleton, J. Destruction of the Field-Induced Density-Wave State in Graphite by Large Magnetic Fields. *Phys. Rev. Lett.* **81,** 5193–5196 (1998).
36. Sun, X.-Q., Zhang, S.-C. & Wang, Z. Helical Spin Order from Topological Dirac and Weyl Semimetals. *Phys. Rev. Lett.* **115,** 076802 (2015).

37. Fauqué, B. *et al.* Two Phase Transitions Induced by a Magnetic Field in Graphite. *Phys. Rev. Lett.* **110,** 266601 (2013).
38. Naughton, M. J. *et al.* Reentrant Field-Induced Spin-Density-Wave Transitions. *Phys. Rev. Lett.* **61,** 621–624 (1988).
39. Kornilov, A. V. *et al.* Novel phases in the field-induced spin-density-wave state in $(TMTSF)_2(PF)_6$. *Phys. Rev. B* **65,** 060404 (2002).
40. Balicas, L., Kriza, G. & Williams, F. I. B. Sign Reversal of the Quantum Hall Number in $(TMTSF)_2(PF)_6$. *Phys. Rev. Lett.* **75,** 2000–2003 (1995).
41. Cooper, J. R. *et al.* Quantized Hall effect and a new field-induced phase transition in the organic superconductor $(TMTSF)_2(PF)_6$. *Phys. Rev. Lett.* **63,** 1984–1987 (1989).
42. Hannahs, S. T., Brooks, J. S., Kang, W., Chiang, L. Y. & Chaikin, P. M. Quantum Hall effect in a bulk crystal. *Phys. Rev. Lett.* **63,** 1988–1991 (1989).
